# GABA-mediated inhibition of human CD4^+^ T cell functions is enhanced by insulin but impaired by high glucose levels

**DOI:** 10.1016/j.ebiom.2024.105217

**Published:** 2024-06-28

**Authors:** Zhe Jin, Hayma Hammoud, Amol Keshavasa Bhandage, Sergiy Vasylyovych Korol, Olivia Trujeque-Ramos, Stasini Koreli, Zhitao Gong, Azasul Islam Chowdhury, Friederike Andrea Sandbaumhüter, Erik Tomas Jansson, Robin Sean Lindsay, Gustaf Christoffersson, Per Erik Andrén, Per-Ola Carlsson, Peter Bergsten, Masood Kamali-Moghaddam, Bryndis Birnir

**Affiliations:** a Department of Medical Cell Biology, Uppsala University, Uppsala, Sweden; b Department of Pharmaceutical Biosciences, Uppsala University, Uppsala, Sweden; c Department of Pharmaceutical Biosciences, Spatial Mass Spectrometry, Science for Life Laboratory, Uppsala University, Uppsala, Sweden; d Department of Immunology, Genetics and Pathology, Science for Life Laboratory, Uppsala University, Uppsala, Sweden

**Keywords:** GABA_A_ receptors, Immunometabolism, Cytokine, Diabetes, Glycolysis, Calcium signaling

## Abstract

**Background:**

γ-aminobutyric acid (GABA), known as the main inhibitory neurotransmitter in the brain, exerts immunomodulatory functions by interaction with immune cells, including T cells. Metabolic programs of T cells are closely linked to their effector functions including proliferation, differentiation, and cytokine production. The physiological molecules glucose and insulin may provide environmental cues and guidance, but whether they coordinate to regulate GABA-mediated T cell immunomodulation is still being examined.

**Methods:**

CD4^+^ T cells that were isolated from blood samples from healthy individuals and from patients with type 1 diabetes (T1D) were activated *in vitro*. We carried out metabolic assays, multiple proximity extension assay (PEA), ELISA, qPCR, immunoblotting, immunofluorescence staining, flow cytometry analysis, MS-based proteomics, as well as electrophysiology and live-cell Ca^2+^ imaging.

**Findings:**

We demonstrate that GABA-mediated reduction of metabolic activity and the release of inflammatory proteins, including IFNγ and IL-10, were abolished in human CD4^+^ T cells from healthy individuals and patients with T1D when the glucose concentration was elevated above levels typically observed in healthy people. Insulin increased GABA_A_ receptor-subunit ρ2 expression, enhanced the GABA_A_ receptors-mediated currents and Ca^2+^ influx. GABA decreased, whereas insulin sustained, hexokinase activity and glycolysis in a glucose concentration-dependent manner.

**Interpretation:**

These findings support that metabolic factors, such as glucose and insulin, influence the GABA-mediated immunomodulation of human primary T cells effector functions.

**Funding:**

The Swedish Children’s Diabetes Foundation, The Swedish Diabetes Foundation, The 10.13039/501100004359Swedish Research Council 2018-02952, EXODIAB, The Ernfors Foundation, The Thurings Foundation and the Science for Life Laboratory.

**Research in context**

**Evidence before this study**

Activation of T lymphocytes is important in fighting various pathogens and cancer, but can also cause problems if erroneously activated such as in autoimmune diseases like type 1 diabetes. Within the body, physiological molecules provide environmental cues and guide the development of T cells. That neurotransmitters can affect immune cells has become apparent within the last decade. Immune cells can encounter neurotransmitters in the brain but also in lymph nodes, in blood and in the pancreatic islets. We and others have shown that the main inhibitory neurotransmitter γ-aminobutyric acid (GABA) can regulate proliferation and release of inflammatory proteins from T cells. In neurons, insulin regulates GABA signalling whereas in pancreatic islets, GABA affects insulin secretion. Antibodies against the glutamic acid decarboxylase, the enzyme synthesising GABA, are detected in type 1 diabetes while glucose concentration in blood is elevated and insulin levels are abnormal in both forms of diabetes (type 1 and 2). Therefore, we investigated how GABA, glucose and insulin modulate T cells immunomodulation.

**Added value of this study**

In this study we demonstrate that the physiological molecules GABA, glucose and insulin all shift the response of T cells. GABA normally puts a brake on T cells proliferation and secretion of inflammatory proteins by activating GABA_A_ receptors channels, enhancing the intracellular Ca^2+^ concentration by influx through CRAC channels and by reducing hexokinase activity but, interestingly, in a glucose-dependent manner. Insulin, in turn, reinforces the GABA-brake on T cells. We elucidate the mechanism of how the GABA-brake comes about and have identified glucose and insulin as physiological molecules that can affect its effectiveness. We reveal that the main obstacle for effective GABA inhibition of T cells functions is high glucose concentration. The ion channels and an intracellular enzyme that participate in the GABA-brake may provide specific pharmaceutical targets. In addition, the results demonstrate that decreasing glucose levels reduces T cells-driven inflammation.

**Implications of all the available evidence**

Prior work has shown that GABA is an important modulator of T cells immune function in health and diabetes. The evidence indicates GABA serving as a homeostatic molecule in the immune system. Nevertheless, at glucose concentrations that may be observed in diabetes, the inhibition normally provided by GABA is overrun and T cells-driven inflammation prevails. The study suggests the importance of decreasing the glucose concentration to maintain the suppressive effect of GABA on inflammation.

## Introduction

Human T cells are a crucial component of the adaptive immune response and provide essential immune protection throughout life. Upon antigen stimulation, naïve T cells undergo clonal expansion and further differentiate into effector cells. During this process, metabolic reprogramming of T cells occurs and is intricately linked to their effector functions.^1^ A variety of soluble molecules including amino acids, other nutrients, and hormones that are present in blood, lymph nodes and tissue microenvironments influence T cell function.^2^, ^3^, ^4^ γ-aminobutyric acid (GABA), is primarily known as an inhibitory neurotransmitter in the central nervous system, but is increasingly recognised as an immunomodulatory molecule.^5^, ^6^, ^7^ We and others have previously shown that GABA inhibits proliferation and cytokine secretion by activating GABA_A_ receptors in CD4^+^ T cells.^8^, ^9^, ^10^ The GABA concentration in the plasma of healthy individuals is around 500 nM and increases somewhat in patients with type 1 diabetes (T1D) or major depression.^10^^,^^11^ In contrast, the GABA content in islets from patients with T1D is decreased relative to healthy individuals.^12^ Glucose is the primary source of energy for activated T cells but, is also utilised for synthesising biomolecules to support the rapid proliferation and differentiation of the cells.^13^^,^^14^ At the time of activation, glucose is mainly transported by glucose transporters that equilibrate glucose concentration across the cell membrane.^3^ The blood glucose concentration varies under physiological and pathophysiological conditions. The typical values for fasting blood glucose concentration in healthy individuals range between 3.9 mM and 5.6 mM glucose. Atypical, pathophysiological glucose levels are observed in diabetes-related hypoglycaemia (<3 mM) or hyperglycaemia (>11 mM).^15^ In human clinical trials, GABA treatment has been shown to be beneficial for glycemic control in patients with T1D.^16^^,^^17^ Insulin, a hormone mainly associated with glucose homeostasis,^15^ also influences T cell function by modulating their metabolism.^18^ We and others have previously demonstrated that GABA signalling regulates hormone secretion in human islets.^12^^,^^19^^,^^20^ However, how the metabolic factors glucose and insulin orchestrate and regulate GABA immunomodulation of T cell functions is still being uncovered.

In the present study, we observe that, in human CD4^+^ T cells, increasing the glucose concentration above typical physiological levels attenuates GABA-mediated inhibition of metabolic activity and release of inflammatory proteins, including IFNγ and IL-10. Furthermore, insulin augments GABA_A_ receptor (GABA_A_R)-mediated currents and Ca^2+^ influx in a manner that is dependent on the glucose concentration. Finally, we show that GABA, insulin and glucose differentially modulate glycolysis in activated CD4^+^ T cells. These results identify glucose and insulin as regulators of GABA immunomodulation impacting human CD4^+^ T cell effector functions.

## Methods

### Study individuals and collection of samples

#### Ethics

Human blood buffy coats and blood samples were obtained from Uppsala University Hospital (Uppsala Akademiska Sjukhuset) following a protocol approved by the Regional Research Ethical Committee (EPN, https://etikprovningsmyndigheten.se) in Uppsala, Sweden (Dnr 2013/347 and Dnr 2014/485). All donors were voluntarily recruited and informed written consent was signed. In total, samples from 165 healthy individuals, 91 men and 74 women with mean age of 26.2 ± 3.2 years and 8 patients with type 1 diabetes aged between 20 and 36 years old were included in the study. The gender was self-reported by study participants. The participants had no signs of infection or inflammation on the day of blood collection. The experimental design is outlined in Supplementary Fig. S1.

### Chemicals and reagents

Drugs, buffers and salts unless otherwise specified were purchased from Sigma–Aldrich/Merck (Steinheim, Germany). GABA (Cat. No. A5835), trans-4-aminocrotonic acid (TACA, Cat. No. 0181/10), muscimol (Cat. No. 0289/1) (1,2,5,6-Tetrahydropyridine-4-yl) methylphosphinic acid (TPMPA, Cat. No.1040/10), YM-58483 (Cat. No. 3939/10) were purchased from Tocris, UK. Urea (Cat.No. 1023430) and NH_4_HCO_3_ (Cat. No. 10207183) were from Acros Organics, Geel, Belgium and trypsin (Cat. No. 13454189) and dimethyl sulfoxide (DMSO) (Cat. No. 10103483) from Thermo Scientific (Waltham, MA). Tris(hydroxymethyl) aminomethane hydrochloride (Tris) (Cat. No. 11420203), LC-MS grade water (Cat. No. 10505904), acetonitrile (Cat. No. 10001334) and formic acid (Cat. No. 10473038) were obtained from Fisher Scientific (Geel, Belgium).

### CD4^+^ T cells isolation and activation

Peripheral blood mononuclear cells (PBMCs) were isolated by density-gradient centrifugation using Ficoll–Paque™ PLUS (Cat. No. GE17-1440-03, Sigma–Aldrich, Sweden). CD4^+^ T cells were then purified from the isolated PBMCs by negative selection using human CD4^+^ T cell isolation kits (Cat. No. 130-096-533, Miltenyi Biotec, Germany) according to the manufacturer's instructions. The culture media (such as, RPMI1640) commonly used for *in vitro* T cell assays usually contain high glucose concentration (>10 mM) and, therefore, are not used. In this study, the freshly isolated CD4^+^ T cells (1x 10^6^/ml) were suspended in RPMI 1640 glucose-free medium (Cat. No. 11879020, Gibco, Fisher Scientific, Sweden) supplemented with 2 mM glutamine (Cat. No. A2916801), 10% heat-inactivated dialyzed fetal bovine serum (Cat. No. A3382001), 100 U/ml penicillin, 10 mg/ml streptomycin (Cat. No. 15140148), and 5 μM β-mercaptoethanol (Cat. No. 31350010, Gibco, Fisher Scientific, Sweden). The activation of the cells was performed in either 96-well (10^5^ cells/well) or 24-well (10^6^ cells/well) plates based on the purpose of the experiment. CD4^+^ T cells were activated with 3 μg/ml plate-bound anti-CD3 (RRID: AB_395736, BD Biosciences, USA) in the presence of different concentrations of glucose (2.8 mM, 5.6 mM, 10 mM and 16.7 mM; Cat. No. G5767, Sigma-Aldrich, Germany) for 66–72 h. Drugs added were based on the type of experiment to be done (see Supplementary Fig. S1).

### MTT (3-(4,5-Dimethylthiazol-2-yl)-2,5-Diphenyltetrazolium Bromide) assay

The metabolic activity of CD4^+^ T cells was assessed in 96-well plates with colorimetric MTT assay. It is commonly used to measure cellular metabolic activity based on mitochondrial dehydrogenase in living cells as an indicator of cell viability and proliferation.^21^ Previous studies^10^ and power analysis using R pwr-package was used to estimate sample size. After 66–72 h of activation, a PBS-soluble tetrazolium dye MTT (Cat. No. 5224/500, Tocris, UK; final concentration 1 mM) was added and incubated for additional 4 h at 37 °C. Thereafter, the cells were centrifuged at 2000 rpm for 10 min to concentrate the insoluble purple formazan pellets. The supernatants were collected and stored at −80 °C for later biomolecules analysis. The formazan crystal pellets were dissolved in DMSO and the plate was read 10 min later using Multiskan FC Microplate Photometer (Thermo Fisher, USA) at 540 nm.

### Multiplex proximity extension assay (PEA)

The supernatants collected at the end of the MTT assay were sent on-ice to the SciLifeLab Affinity Proteomics Unit in Uppsala, Sweden, for analysis. Inflammatory proteins were measured using high-throughput multiplex PEA with an Olink Target 96 Inflammatory panel (Cat. No. 95302, OlinkProteomics, Uppsala, Sweden). PEA measures simultaneously 92 proteins/biomolecules in different sample matrices where it combines a dual-detection immunoassay and quantitative PCR. Normalised protein expression (NPX) values, an arbitrary unit on the log_2_ scale, were used to present the data. A protein expression (NPX value) is considered acceptable if the value is above the limit of detection (LOD) for each particular protein. Proteins that were detected in more than 75% of the samples were analysed in this study: 59 or 53 out of 92 proteins had detectable levels, in samples from healthy individuals and patients with T1D, respectively. Data for resting, activated and GABA-treated activated CD4^+^ T cells supernatants were analysed separately. The biomolecules in the supernatants were assessed for each donor at each glucose concentration. The data were computed, processed and analysed with support from NBIS (National Bioinformatics Infrastructure Sweden). The log_2_FC (FC = fold change) is the average increase in NPX in GABA as compared to activated alone, this value is both estimated based on the linear mixed effects model and computed as the mean over the GABA-activated difference and plotted in heatmaps. Paired t-test was used followed by multiple testing correction using false discovery rate (FDR) method (Two-stage step-up method of Benjamini, Krieger and Yekutieli) with a threshold 12%.

### Determination of IFNγ and IL-10 concentrations in supernatants

The concentrations of IFNγ and IL-10 were measured with enzyme-linked immunosorbent assay (ELISA). The supernatants were diluted when necessary to be in the optimal linear optical range and analysed using commercial kits for IFNγ (Human IFN-gamma DuoSet ELISA, Cat. No. DY285B, R&D Systems, USA and ELISA MAX™ Deluxe Set Human IFN-gamma, Cat. No. 430104, BioLegend, UK), and IL-10 (Cat. No. 430604, Biolegend, UK) according to manufacturers’ instructions. Optical densities were measured using FC Microplate Photometer (Thermo Fisher, USA) at 450 nm, and 540 nm was used as a correction wavelength. The levels of IFNγ or IL-10 were normalised to controls (activated CD4^+^ T cells in the absence of drugs).

### Total RNA and protein extraction

Total RNA and protein were extracted from activated CD4^+^ T cells, in the absence or presence of 500 nM GABA, using RNA/Protein Purification Plus Kit (Cat. No. 48200, Norgen Biotek, Ontario, Canada). During the extraction process, total RNA was treated with RNase-Free DNase I Kit (Cat. No. 25710, Norgen Biotek, Ontario, Canada) to eliminate contamination of genomic DNA. The extracted proteins were quantified using DC protein assay kit (Cat. No. 5000112, Bio-Rad, USA), and immediately frozen at −80 °C.

### Real-time quantitative reverse transcription (qPCR)

The cDNA was synthesised using SuperScript reverse transcriptase IV (Cat. No. 18090010, Invitrogen, Thermo Scientific, USA) in a 20 μl reaction following the standard protocol from manufacturer. The qPCR reaction was then performed using qPCRBIO SYBR or Probe Mix low-ROX (Cat. No. PB20.11-05, PB20.21-05, PCR Biosystems, UK), and gene-specific primers (Supplementary Table S4) or Taqman probes (*GABRR2* Hs00266703_m1; *IPO8* Hs00914057_m1, ThermoFisher Scientific, USA). Changes in the abundance of each transcript were normalised to the expression of reference genes (*IPO8*). Relative expression levels were expressed as ΔCt (Ct, cycle threshold) or calculated by normalising to non-treated activated CD4^+^ T cells obtained from the same donor at the same glucose concentration using 2^-ΔΔCt^ method. All samples were run in duplicate. The real-time qPCR amplification was performed in 10 μl reaction mix in a 384-well plate using a QuantStudio™ 5 instrument (ThermoFisher Scientific). All runs started with an initial denaturation step of 2 min at 95 °C, followed by 45 cycles of 95 °C for 5 s and 60 °C for 30 s.

### Mass spectrometry (MS)-based bottom-up proteomics

The lysates were purified and tryptic digested for MS-based bottom-up proteomic analysis using Filter Aided Sample Preparation (FASP).^22^^,^^23^ Briefly, 20 μg of total protein were transferred onto centrifugal filter units (Cat. No. MRCF0R030, Microcon-30 kDa; Merck, Darmstadt, Germany) and washed with a buffer containing 8 M urea and 100 mM Tris (pH 8.5). The washing step was repeated after reduction with 8 mM dithiothreitol (DTT), alkylation with 50 mM iodoacetamide (IAA) and removal excess of IAA with 8 mM DTT. Before tryptic digestion (enzyme-to-protein ratio 1:50 (w/w)) the filter was washed three times with NH_4_HCO_3_. After incubation in a wet chamber at 37 °C for 16 h the resulting peptides were washed twice from the filter by adding 50 mM NH_4_HCO_3_. Trifluoroacetic acid was then added to a final concentration of 1% (v/v) and the samples were dried at 45 °C. Finally, the samples were reconstituted in 3% acetonitrile and 0.1% formic acid in water to a final concentration of 150 ng protein/μl. The tryptic peptides were analysed on a nanoAcquity UPLC system equipped with a C18, 5 μm, 180 μm × 20 mm trap column and an HSS-T3 C18 1.8 μm, 75 μm × 250 mm analytical column (Waters Corporation, Manchester, UK) coupled to a Synapt G2 Si HDMS mass spectrometer with an electrospray ionisation source (Waters Corporation, Manchester, UK). The UDSME approach with positive ionisation was used.^22^^,^^23^ Mobile phase A contained 3% DMSO and 0.1% formic acid in water and mobile phase B 3% DMSO and 0.1% formic acid in acetonitrile. After loading 300 ng of protein on the column in trapping mode, peptide separation was performed with a gradient run from 3 to 40% (v/v) mobile phase B over 120 min. The flow rate was set to 0.3 μl/min and the column oven was set to 40 °C. Method performance was controlled with a commercially available HeLa digest (Cat. No. 88328, Thermo Scientific, Waltham, MA). UPLC-MS data processing and label-free quantification: ProteinLynx Global Server (version 3.0.3, Waters Corporation, Milford, MA) was used for raw data processing. With FDR of 10%, a database search against a randomised UniProt human database (Uni-ProtKB version 14/01/2020) was performed. Carbamidomethyl cysteine was set as fixed modification, acetyl lysine, C-terminal amidation, asparagine deamidation, glutamine deamidation and methionine oxidation as variable modifications and trypsin as digest reagent. Minimum peptide matches per protein were 2 and minimum fragment ion matches per peptide and protein were 1 and 3, respectively. For label-free quantification analysis, the identified proteins were further processed using ISOQuant 1.8 as described elsewhere.^22^ Following the TOP3 quantification approach the average intensity of the three most intense peptides of each protein were used for relative protein quantification.

### Immunoblotting

CD4^+^ T cells were lysed in RIPA buffer (Cat. No. R0278, Sigma–Aldrich, Germany) containing protease inhibitor cocktail (Cat. No. 11836153 001, Roche) and a phosphatase inhibitor sodium orthovanadate (Cat. No. S6508, Sigma–Aldrich). Proteins were heated at 100 °C for 5 min in SDS loading buffer (275 mM Tris-HCL, 9% SDS, 50% glycerol, 0.03% bromophenol blue) containing 9% beta-mercaptoethanol and separated by Mini-Protean TGX gel 4–15% (Cat. No. #4568084, 4561035, BioRad, stain-free gels) and blotted onto Amersham Hybond-P PVDF membrane (Cat. No. GE10600023, Sigma-Aldrich). Membranes were blocked with blocking buffer (Cat. No. 12010020, EveryBlot, BioRad) for 20 min at room temperature (RT) and incubated overnight at 4 °C with the following primary antibodies: HK1 (GenTex, Cat. No. GTX105248, RRID: AB_1950492, 1:3000), GABRR2 (Invitrogen, PA5-41008, 1:1000, RRID: AB_2609056) and NFATs; NFAT1 (GeneTex, GTX127932, RRID: AB_2885655, 1:2000), NFAT2 (Novus Biologicals, NB100-56732, RRID: AB_838619, 1:1000), or NFAT4 (GeneTex, GTX133744, RRID: AB_2887088, 1:2000). After immunoblotting, membranes were washed in TBST and incubated with Peroxidase-conjugated AffiniPure secondary antibodies (Jackson ImmunoResearch, 111-035-045, RRID: AB_2337938, 1:5000). Protein detection was done using chemiluminescent ECL reagent (Thermo scientific, Cat. No. 32209). Quantitative densiometric analysis of the immunoblotted bands was performed using Image Labs software (BioRad Laboratories, V6.0.0) and data were normalised for total proteins of each sample using stain-free technique.

### Immunofluorescence staining

CD4^+^ T cells were harvested after 72 h of anti-CD3 activation on cytoslides (Thermo scientific, Cat. No. 5991056) using a Shandon cytospin centrifuge (400 rpm for 4 min at RT). After drying, cells were fixed with 4% PFA in PBS for 15 min and washed 3 times, followed by membrane permeabilisation with 0.1% Triton-X for 5 min. Non-specific binding was blocked with 5% bovine serum albumin and 10% donkey serum (Jackson ImmunoResearch, 017-000121) in 1X PBS for 60 min. Cells were then washed and incubated with a primary antibody rabbit anti-GABRR2 (Invitrogen, PA5-41008, RRID: AB_2609056, 1:600) diluted in blocking solution overnight at 4 °C followed by incubation with an AlexaFluor 488-conjugated AffiniPure Donkey Anti-Rabbit IgG secondary antibody (Jackson ImmunoResearch, 711-545-152, RRID: AB_2313584, 1:500) for 1h at RT. Finally, cells were washed again and counterstained with DAPI for 5 min and slides were mounted in ProLongTM Gold antifade reagent (Cat. No. P36930, Invitrogen). Images were acquired using confocal microscopy (LSM700, Zeiss) with 63X objective. For statistics, fluorescence intensities of ≥40 cells from 3 donors were quantified using FiJi ImageJ (NIH) software.^24^

### Flow cytometry methods

Following CD4^+^ T cell activation and treatment, single cell suspensions were washed in PBS and stained with Fixable Live/Dead Aqua (Cat. No. L34966, Invitrogen) according to manufacture's instructions. Following Live/Dead staining cells were washed into FACS buffer (PBS containing 0.5% BSA and 2 mM EDTA. Cells were then stained in a master mix containing Brilliant Violet Buffer (Cat. No. 563794, BD Biosciences), Human F_C_ block (Cat. No. 564219, BD Bioscience) and antibodies targeting surface antigens: anti-CD3 BV421 (Clone OKT3, Cat. No. 317344, RRID: AB_2565848, Biolegend), anti-CD4 AF488 (Clone OKT4, Cat. No. 317420, RRID: AB_571938, Biolegend), Anti-CD8a APC (Clone RPA-T8, Cat. No. A18611, RRID AB_2535401, Invitrogen), anti-HLA-DR PE-Fire640 (Clone L243, Cat. No. 307676, RRID: AB_2876604, Biolegend), anti-CD25 PE (Clone BC96, Cat. No. 302606, RRID: AB_314275, Biolegend), anti-CD57 BV785 (QA17A04, Cat. No. 393330, RRID: AB_2860967, Biolegend), anti-PD-1 BV650 (EH12.2H7, Cat. No. 329950, RRID: AB_2566362, Biolegend), and anti-CTLA-4 BV605 (Clone BNI3, Cat. No. 369610, RRID: AB_2632778, Biolegend). Cells were incubated for 30 min at 4 °C followed by washing with FACS buffer. Cells were then fixed and permeabilised for intracellular staining using Foxp3/Transcription Factor Staining Buffer (Cat. No. 00-5523-00, Invitrogen) for 30 min prior to intracellular staining in Permeabilization buffer (Cat. No. 00-8333-56, Invitrogen), anti-FoxP3 PE-Cy7 (Clone PCH101, Cat. No. 25-4776-42, RRID: AB_10804638, Invitrogen) and anti-Ki-67 APC-eFluor780 (Clone SolA15, Cat. No. 47-5698-82, RRID: AB_2688065, Invitrogen). Following washing samples were analysed on a Cytek Northern Lights spectral flow cytometer equipped with 3 lasers. Following data acquisition sample data was unmixed using the SpectroFlow software (Cytek) and analysed using Flowjo software (BD Bioscience). Gates were set based on fluorescence minus one (surface antigens) or isotype controls (intracellular antigens).

### *In vitro* hexokinase activity assay

Hexokinase activity was measured at RT indirectly as a readout of NADH production, a by-product of the reaction converting glucose-6-phosphate to 6-phosphogluconolacton by the enzyme glucose-6-phosphate dehydrogenase (GPDH). The cells were harvested, centrifugated and washed twice with PBS. Washed cells were resuspended in 1 ml assay buffer solution, on ice, included in the hexokinase enzymatic assay kit (Bio Vision, Cat. No. K789-100). After centrifugation at 4 °C for 5 min, the resulting supernatant was used as the final cell extract for the enzymatic assay.

### Electrophysiological recordings

The electrophysiological recordings from the isolated CD4^+^ T cells were done using the perforated whole-cell patch-clamp configuration. In order to keep the intracellular chloride concentration intact, gramicidin was used to obtain the perforated configuration. Gramicidin (Cat. No. G5002, Sigma-Aldrich) was diluted in DMSO at the stock concentration 2 mg/ml and the final concentration of the gramicidin in the pipette solution was 2.6 μg/ml. The pipette solution with the gramicidin was protected from light, kept cold and used within 2 h after the preparation. After making the cell-attached configuration, the access resistance was monitored until characteristic slow and low-amplitude capacitance transients were observed and the access resistance decreased from GΩ range to the range of tens of MΩ. The extracellular solution (in mM) contained: 157 NaCl, 4.5 KCl, 0.5 CaCl_2_, 1 MgCl_2_, 5 HEPES and 5.6 or 16.7 glucose (pH 7.4, adjusted with NaOH). The pipette solution consisted of (mM): 149 KCl, 2 CaCl_2_, 1 MgCl_2_, 1 NaCl, 10 HEPES, 5.6 or 16.7 glucose (pH 7.3 adjusted with KOH). The single-channel recordings were done in the whole-cell patch-clamp configuration.^20^ The extracellular solution (in mM) contained: 137 NaCl, 3 KCl, 1 CsCl, 0.02 CaCl_2_, 2 MgCl_2_, 5 HEPES and 5.5 glucose (pH 7.4, adjusted with NaOH). The pipette solution consisted of (mM): 137 CsCl, 4 KCl, 0.01 CaCl_2_, 0.5 MgCl_2_, 5 HEPES, 5 EGTA, 2 Na_2_ATP (pH 7.3 adjusted with NaOH). The patch-clamp pipettes were made from borosilicate glass and had a resistance of 10–14 MΩ when filled with the pipette solution and immersed in the extracellular solution. Recordings were done using Axopatch 200B amplifier, filtered at 2 kHz and digitised on-line at 10 kHz using an analog-to-digital converter. To record the electrophysiological data Clampex 10.5 (Molecular Devices, San Jose, CA, USA) software was used. In the perforated patch-clamp configuration the currents were recorded at +30 mV or by a ramp protocol, where the potential was changed from −80 to +80 mV in 1 s, the holding potential (Vh) was kept −40 mV. The single sweep duration was 20 s with a break between sweeps 5 s. After application of a drug, the chamber was perfused 2–6 min with extracellular solution only, before next application of a drug. Currents in the absence of drugs or in the presence of picrotoxin were subtracted from the test-drug current response. For single-channel recordings,^20^ currents were activated by perfusing 500 nM GABA into the recording chamber. The holding potential was changed in steps from −80 to +80 mV to record the current–voltage (IV) relationship of the channels.

### Live cell time-lapse real-time Ca^2+^ imaging

Cells were loaded with a calcium indicator, 3 μM Fluo-8 AM (Cat. No. 21082, AAT Bioquest, Pleasanton, CA) for 15 min at 37 °C and seeded on 5% 3-aminopropyltriethoxysilane-coated coverslip (Cat. No. A3648, Sigma Aldrich) for 15 min at 37 °C. Time lapse images were acquired by confocal microscopy (LSM700, Zeiss) with 40X objective at an interval of 1–1.5 s per image. Drugs were diluted in RPMI 1640 without phenol red (Cat. No. 32404014, A2494201, Invitrogen) and perfused at indicated time and concentration by a peristaltic pump. After application of each drug, cells were perfused with the extracellular solution (RPMI 1640) before next application. Area for single cells was marked and absolute fluorescence intensity values (F) were extracted using ZEN software. Relative intensity (F/Fmedian) for each cell was calculated for every recorded image and plotted against time. Further, maximum relative intensity of the cells under drug application time is considered as drug's response. Data is represented for all cells and as box-whiskers plot.

### Metabolism assay

Isolated CD4^+^ T cells were plated in a XFe96 cell plates that were coated with Cell-Tak (Cat. No. 354240, Corning, New York, USA) according to manufacturer's protocol. The metabolic function of activated CD4^+^ T cells cultured for 3 days *in vitro* was analysed by measuring the extracellular acidification rate (ECAR) using an XFe96 extracellular flux analyser (Seahorse Bioscience). The cells were kept in XF media (Seahorse Bioscience) supplemented with 5 mM glucose, and subjected to glycolysis stress-test protocol using sequential injection of 25 mM glucose, 2 μM oligomycin, and finally 15 mM 2-deoxyglucose (2DG). Measurements for each experiment were conducted at least in triplicate and glycolysis, glycolytic capacity, and glycolytic reserve were calculated and values normalised to protein content.^25^^,^^26^

### Statistics

Statistical analysis was performed using GraphPad Prism 9 (GraphPad Software Inc., La Jolla, CA, Version 9.3.1). Cellular metabolic activity, cytokine levels, mRNA, protein expression and ECAR of CD4^+^ T cells in the presence of indicated drugs were normalised to controls (activated cells without drug treatment) from each donor and for each glucose concentration. Shapiro–Wilk test was used to assess the data normality. Values more than 1.5 times the interquartile range (IQR) from the quartiles are defined as outliers (Tukey's methods) and presented outside whiskers in the box-and-whisker plot. For comparing two groups, paired t-test or Wilcoxon test for paired data, or Mann–Whitney test for unpaired data was performed. A comparison between multiple paired groups was performed using repeated measures one-way or two-way ANOVA or mixed-effects model followed by Tukey or Fisher's LSD multiple comparisons tests. Unpaired multiple-group comparison was performed using ordinary one-way ANOVA followed by Tukey test or Friedman test followed by Dunn's test. Changes in protein levels in MS data were analysed using Quantitative, multi-dataset Pathway Analysis (ReactomeGSA, https://reactome.org/PathwayBrowser/#TOOL=AT) and volcano plot was subsequently generated. Spearman correlation coefficients are annotated and linear regression line is plotted using "lm" package in R 4.0.0. The statistical significance level was set to 0.05.

### Role of funders

The funders had no role in the study design, data collection, data analysis and interpretation, or the decision to prepare or publish this manuscript.

## Results

### GABA inhibition of activated T cell function is affected by glucose

**Fig. 1:**
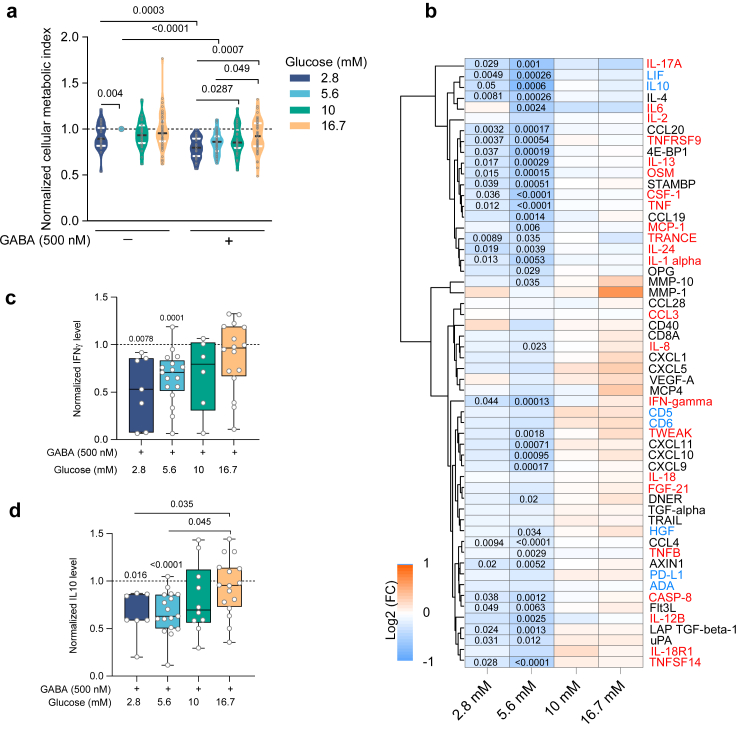
**GABA effects on metabolic activity and cytokine release are glucose concentration-dependent in activated CD4**^**+**^**T cells**. **a)** Violin plots show the cellular metabolic activity of activated CD4^+^ T cells as measured by MTT assay, in the absence and presence of GABA at different glucose concentrations: 2.8 (N = 32), 5.6 (N = 51), 10 (N = 32) and 16.7 mM (N = 51). Horizontal black and white lines indicate the median and quartiles, respectively. Grey circles indicate individual donors. Data were normalised to values of activated cells in the absence of GABA at 5.6 mM glucose concentration. **b)** Heatmap and hierarchical clustering of inflammatory-related proteins by PEA 72 h post-stimulation of CD4^+^ T cells, at different glucose concentrations (N = 17). Data represent the mean log_2_(fold change) for samples cultured in presence and compared to absence of GABA. **c, d)** Box plots show GABA inhibition of IFNγ **(c)** and IL-10 **(d)** release from CD4^+^ T cells from healthy individuals, as measured by ELISA, glucose concentrations (mM): 2.8 (N = 7; 7), 5.6 (N = 17; 17), 10 (N = 6; 10) and 16.7 (N = 16; 16) in **c** and **d**, respectively. Data were normalised to values of activated cells in the absence of GABA at each glucose concentration. Box and whisker: box as 25–75 percentiles, whiskers determined with Tukey's method, black lines in the boxes as median. Statistics: Repeated measures two-way ANOVA (mixed model) followed by Tukey for multiple comparisons (**a**), dependent t-test followed by multiple testing correction using false discovery rate (FDR) (**b**), Wilcoxon test for comparing paired raw values in the absence or presence of GABA at each glucose concentration, and mixed-effects analysis for normalised values among different glucose concentrations (**c, d**). P-values are indicated in the figures. **N**: donors.

**Fig. 2:**
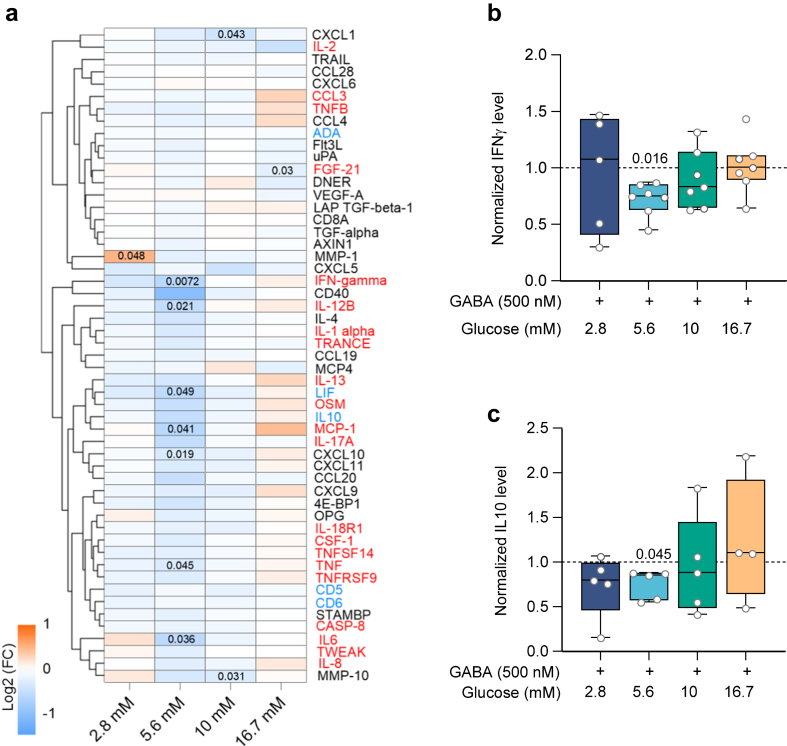
**GABA effects on cytokine release in activated CD4**^**+**^**T cells from patients with type 1 diabetes (T1D)**. **a)** Heatmap and hierarchical clustering of inflammatory-related proteins by PEA 72 h post-stimulation of CD4^+^ T cells, at different glucose concentrations (N = 8). Data represent the mean log_2_(fold change) for samples cultured in presence and compared to absence of GABA. **b, c)** Box plots show GABA inhibition of IFNγ **(b)** and IL-10 **(c)** release from CD4^+^ T cells from patients with T1D, as measured by ELISA, glucose concentrations (mM): 2.8 (N = 5; 5), 5.6 (N = 7; 5), 10 (N = 7; 5) and 16.7 (N = 7; 4) in **b** and **c**, respectively. Data were normalised to values of activated cells in the absence of GABA at each glucose concentration. Box and whisker: box as 25–75 percentiles, whiskers determined with Tukey's method, black lines in the boxes as median. Statistics: Dependent t-test or Wilcoxon test for comparing paired raw values in the absence or presence of GABA at each glucose concentration (**a, b, c**), and mixed-effects analysis for normalised values among different glucose concentrations (**b, c**). P-values are indicated in the figures. **N**: donors.

Physiological blood glucose concentration 2 h after a meal, is normally maintained around 5.6 mM in healthy individuals, while it is higher in diseases such as diabetes (>7 mM)^15^ or Covid-19.^27^ The physiological GABA concentration in human plasma is about 500 nM^10^^,^^28^ and provides a homeostatic inhibition on T cells.^10^ Importantly, metabolic activity of T cells is closely related to their function.^1^ Therefore, we examined effects of four glucose concentrations on the GABA inhibition of the metabolic activity of CD4^+^ T cells (N = 32–51 individuals; Fig. 1a; Supplementary Fig. S2a, Supplementary Table S1). At glucose concentrations higher (10 mM and 16.7 mM) than the typical physiological level (5.6 mM), the inhibition by GABA (500 nM) was abolished (Fig. 1a). In absence of GABA, the metabolic activity of activated CD4^+^ T cells varied somewhat for the different glucose concentrations. In the low-physiological glucose, 74% of the samples were inhibited by GABA (Supplementary Fig. S2b and c) whereas at the higher (10 mM and 16.7 mM) glucose concentrations, samples from these same individuals were not inhibited by GABA (Supplementary Fig. S2c). The remaining 26% of the samples that were obtained from different individuals were not inhibited by GABA at any glucose concentration (Supplementary Fig. S2d). Intrigued by these results, we sought to determine whether glucose affected other immunological functions regulated by GABA. Since glycolysis feeds substrates into anabolic metabolism, we studied if glucose modulated the GABA-inhibition of the release of biomolecules from CD4^+^ T cells cultured in low, physiological or high glucose. In each sample, 92 inflammatory-related biomolecules commonly associated with inflammation were measured using Olink Target Inflammation protein panel, which is based on the multiplex proximity extension assay (PEA) technology. A total of 59 different proteins were detected and included 23 pro- and 7 anti-inflammatory proteins identified by red and blue lettering (Fig. 1b), respectively. Similar to the results from the metabolic activity, the GABA-inhibition of proteins released from CD4^+^ T cells was glucose concentration-dependent (Fig. 1b). The GABA inhibition was optimal at the physiological glucose concentration (5.6 mM), where GABA decreased the release of 37 proteins, including 17 pro- and 3 anti-inflammatory proteins. In contrast, GABA did not affect release of proteins in either 10 mM nor 16.7 mM glucose (Fig. 1b). We further performed ELISA using CD4^+^ T cells culture supernatants from samples from healthy individuals, for two cytokines; interferon gamma (IFNγ), a pro-inflammatory and the principal cytokine of CD4^+^ T helper1 cells (Th1) and an anti-inflammatory protein interleukin-10 (IL-10), the principal cytokine for CD4^+^ T helper2 (Th2) and regulatory T (Treg) cells (Fig. 1c and d). GABA-mediated inhibition of IFNγ and IL-10 release was observed in low to physiological glucose (2.8 and 5.6 mM), but not in high glucose (10 and 16.7 mM) in samples from healthy individuals (Fig. 1c and d). In the absence of GABA, increasing the glucose concentration to 16.7 mM alone enhanced the release of IFNγ (Supplementary Fig. S2e) but not IL-10 in samples from healthy individuals (Supplementary Fig. S2f). In contrast, a total of 53 different proteins, including 21 pro- and 5 anti-inflammatory proteins, were detected in the PEA assay in samples from patients with T1D (N = 8) but only 10 were significantly reduced by GABA (Fig. 2a), the majority at 5.6 mM glucose and included 5 pro- and one anti-inflammatory proteins. We further examined if correlation might be found between duration of diabetes or HbA1c levels in the samples from the patients with T1D and the proteins detected by PEA at each glucose concentration (Supplementary Fig. S3). For our relatively few samples, correlations are difficult to assess but, there is a trend for e.g., the levels of IFNγ and IL-10 at 5.6 mM glucose to correlate with diabetes duration and Hb1c that was not detected at the other glucose concentrations. We then examined by ELISA the culture supernatants from the CD4^+^ T cells from the patients with T1D (N = 7) (Fig. 2b and c). GABA significantly reduced the release of IFNγ and IL-10 from CD4^+^ T cells from the patients with T1D but only at the 5.6 mM glucose concentration (Fig. 2b and c). Thus, the results suggest that GABA-dependent regulation of CD4^+^ T cells is linked to the extracellular glucose concentration and is altered in T1D.

### Insulin enhances the GABA-activated currents and modulates GABA_A_ receptor expression in CD4^+^ T cells

**Fig. 3:**
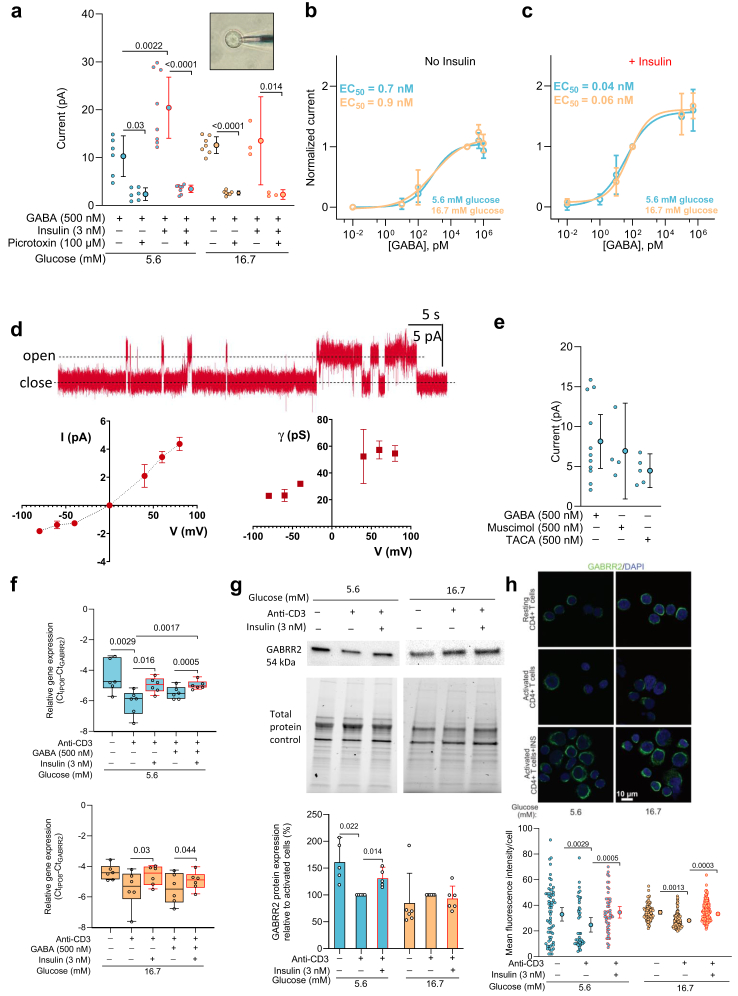
**Insulin enhances GABA-activated GABA**_**A**_**R currents and regulates GABA**_**A**_**R expression in activated CD4**^**+**^**T cells**. **a)** Saturating GABA-activated whole-cell currents (at +30 mV) in the absence or presence of a GABA_A_R antagonist picrotoxin (PTX) in activated CD4^+^ T cells without or treated with insulin (n = 3–8, N = 11). Insert: a patch-pipette attached to an activated CD4^+^ T cell. **b, c)** GABA dose–response curves and the calculated half maximal effective concentration (EC_50_) in cells without (**b**) or treated with (**c**) insulin. The currents were normalised to 100 nM (**b**) or 100 pM (**c**), GABA responses (n = 3–11, N = 27). Curve fitting: nonlinear regression with sigmoidal polynomial (4 PL) model. **d)** Representative single-channel currents evoked by GABA (500 nM, +60 mV) in an activated CD4^+^ T cell. In symmetrical chloride solutions, the channels current-voltage relationship (lower left panel) is outwardly rectifying, and the channel conductance is greater at depolarised as compared to hyperpolarised potentials (lower right panel). **e)** Whole-cell currents (at +30 mV) evoked by GABA_A_R agonists GABA (n = 11, N = 9), muscimol (n = 4, N = 3) or TACA (n = 5, N = 4), in activated CD4^+^ T cells in 5.6 mM glucose. **f)** Relative expression of *GABRR2* mRNA (ΔCt) in resting and activated cells in 5.6 mM or 16.7 mM glucose (N = 6). Box-whisker plots (black without and red with insulin: box as 25–75 percentiles, whiskers determined with Tukey's method, black lines in the boxes as median. **g)** Representative immunoblot images and relative expression of GABRR2 protein (N = 5). Target band volumes after total protein normalisation were further normalised to values of activated cells in the absence of GABA at each glucose concentration. **h)** Representative images from immunofluorescence co-staining of GABRR2 (green) and DAPI (blue) in CD4^+^ T cells in 5.6 mM or 16.7 mM glucose. Bar graphs show quantification of GABRR2 fluorescence intensity in CD4^+^ T cells (n ≥ 40 cells/group, N = 3). In experiments with insulin, 48-h post-activation insulin was added for 24-h. Data represent mean with 5–95 percentile in (**a, e, g, h**) and mean ± SD in (**b, c, d**). Statistics: ordinary (**a, e, h**) or repeated measures **(f)** one-way ANOVA followed by Tukey multiple comparison test; one sample t-test when compared to activated cell group (**g**). **n**: cells, **N**: donors.

Glucose homeostasis is influenced by a variety of hormones, with insulin being one of the key players. Interestingly, at least in excitable cells, insulin is also a regulator of GABA signalling.^29^, ^30^, ^31^ The insulin receptor is not expressed in resting T cells but is prominent at 48 h post-activation.^18^ Therefore, after 48 h activation, we incubated CD4^+^ T cells with insulin at physiologically relevant concentration (3 nM) and where unspecific activation of other receptors types does not take place.^18^^,^^31^ GABA activates two types of receptors in cell membranes; the GABA_A_ receptor (GABA_A_R), a pentameric chloride ion channel, that opens when GABA binds to the receptor and the GABA_B_ receptor (GABA_B_R), a G-protein coupled receptor, that is formed as a dimer**.** To date, only one of the two GABA_B_ isoforms required for function has been detected in immune cells, whereas several GABA_A_ receptor subunits are identified in T cells.^4^^,^^10^ We measured the GABA-activated GABA_A_R whole-cell currents in intact activated CD4^+^ cells using perforated patch-clamp electrophysiology (Fig. 3a, b, c, e). GABA was acutely applied to the cells and the cells had not been exposed to GABA before. Since the intracellular environment is unchanged, the intracellular signalling that may take place is expected to function normally. The saturating, 500 nM GABA-activated GABA_A_R-mediated whole-cell currents were blocked by the GABA_A_R antagonist picrotoxin. Picrotoxin is an open channel blocker of GABA_A_R. These maximal whole-cell current responses were only enhanced by insulin at 5.6 mM but not at 16.7 mM glucose (Fig. 3a), whereas the apparent GABA affinity (EC_50_) of GABA_A_R was similar in 5.6 mM and 16.7 mM glucose, 0.7 nM and 0.9 nM GABA, respectively (Fig. 3b). Insulin then shifted the EC_50_ values more than 10-fold, to 0.04 nM and 0.06 nM GABA (Fig. 3c), for the two glucose concentrations, respectively. These results suggest that insulin may increase the GABA_A_R plasma membrane surface number in 5.6 mM glucose but enhances the GABA affinity of GABA_A_R in CD4^+^ T cells similarly at both glucose concentrations.

The single-channel currents activated by 500 nM GABA showed outward rectification, and the channels were high-conductance at depolarised potentials (Fig. 3d), similar to hippocampal^32^ GABA_A_R channels but the conductance was larger than reported for rat retinal^33^ or expressed^34^ ρ-containing GABA_A_R channels. The GABA_A_R agonists muscimol and TACA evoked whole-cell currents similar to GABA (Fig. 3e). The perforated-patch currents were stable (Supplementary Fig. S4b) and reversed at −37 mV (Supplementary Fig. S4a), close to the calculated reversal potential for chloride (E_Cl_ = −39 mV). By regulating the membrane potential these very sensitive GABA_A_R channels may affect the activity of other ion channels or flow of ions across the cell membrane.^35^

Among the 19 GABA_A_R subunits, the GABA_A_R ρ2 (*GABRR2*) is a highly expressed subunit in peripheral blood mononuclear cells.^10^ We therefore examined the ρ2 mRNA and protein expression levels in CD4^+^ T cells. In 5.6 mM glucose, activation of CD4^+^ T cells decreased the expression of the *GABRR2* transcript relative to resting cells, whereas insulin increased the expression again of the *GABRR2* in the activated cells (Fig. 3f). In 16.7 mM glucose, the level of expression of *GABRR2* was similar between activated and resting but insulin appeared to increase the level somewhat (Fig. 3f). Similar trend was observed for GABRR2 protein expression identified by ρ2-specific antibody in samples from homogenised CD4^+^ T cells (Fig. 3g) and where the GABRR2/ρ2 was detected by fluorescent labelling in the CD4^+^ T cells (Fig. 3h). Taken together, the data show that insulin potentiates GABA signalling in activated CD4^+^ T cells by enhancing ρ2-containing GABA_A_R expressed in the cells.

### Insulin enhances GABA-activated Ca^2+^ entry in CD4^+^ T cells

**Fig. 4:**
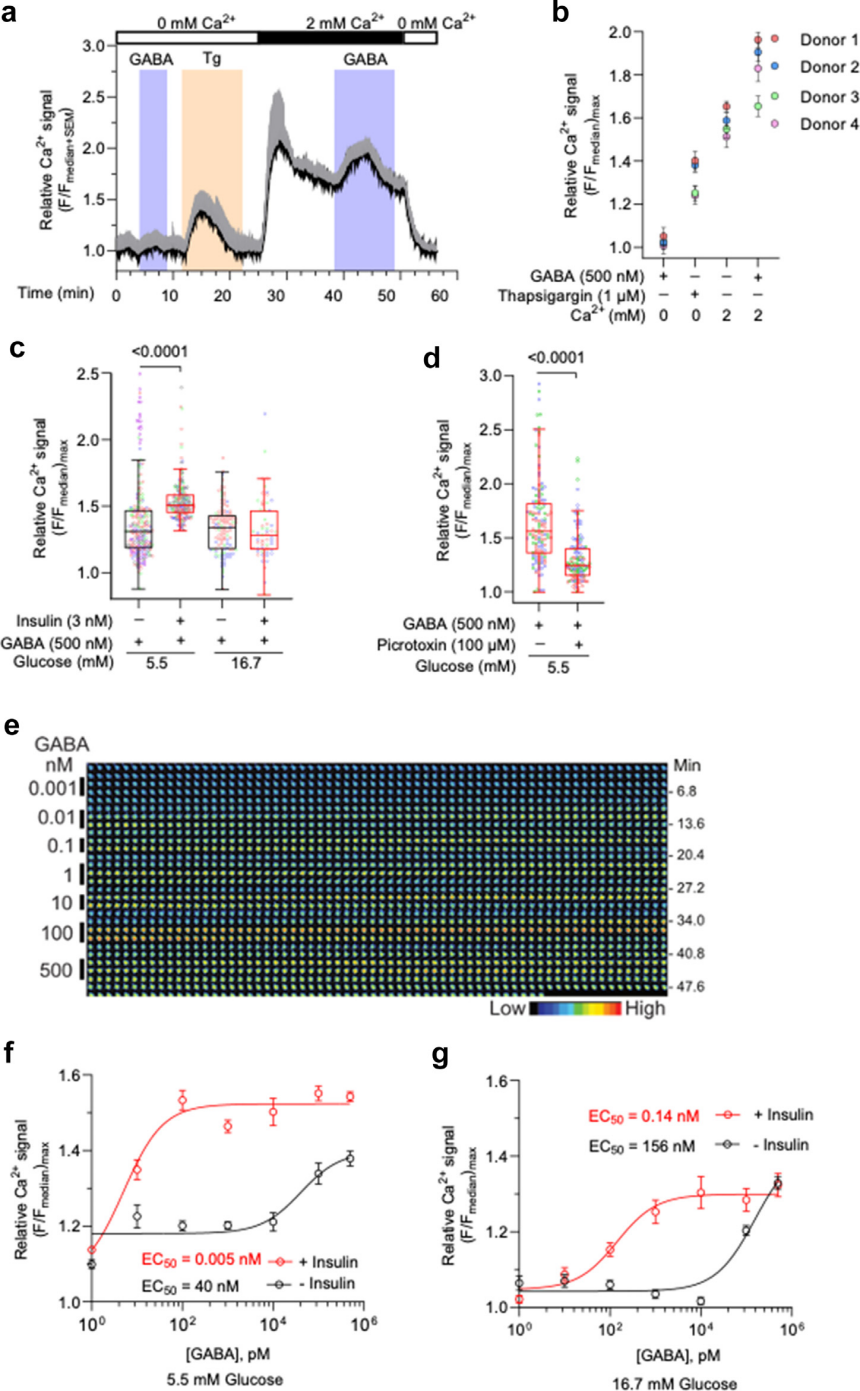
**Insulin elevates GABA-activated Ca**^**2+**^**entry in human CD4**^**+**^**T cells**. **a)** Mean Ca^2+^ signal intensity trace (black line) and SD (gray whiskers) recorded 72 h after activation of cells (n = 14). Coloured bars: Perfusion time of drugs (GABA 500 nM or thapsigargin, Tg 1 μM) in 0 or 2 mM Ca^2+^-containing perfusion medium. **b**) Relative Ca^2+^ signal (mean ± SD) recorded in activated CD4^+^ T cells (n = 14, 20, 41, 47, N = 4). **c)** GABA-activated relative Ca^2+^ signal recorded in activated CD4^+^ T cells (n = 58–232, N = 3–5) without or treated with insulin. **d)** GABA-activated relative Ca^2+^ signal in activated CD4^+^ T cells (n = 131, N = 3) in absence or presence of picrotoxin, with insulin. **e)** Representative time-lapsed micrographs of a live Ca^2+^ imaged cell (16.7 mM glucose, with insulin). GABA concentrations (vertical bars on the left) were sequentially perfused (time indicated as min on the right) and inter-spaced with medium applications. Colour scale: Relative Ca^2+^ fluorescence intensity. **f, g)** GABA dose-response relationship and the calculated half maximal effective GABA concentration (EC_50_) for GABA-induced Ca^2+^ signals in activated CD4^+^ T cells without or treated with insulin in 5.5 mM (**f**) or 16.7 mM (**g**) glucose (n = 63–279, N = 3–8), see also Supplementary Fig. S4f. Curve fitting: nonlinear regression with sigmoidal polynomial (3 PL) model. In (**c, d**), individual data point represents relative Ca^2+^ signal recorded from each cell and is color-coded based on donors and box-whisker plots with Tukey's method. In experiments with insulin, 48-h post-activation insulin was added for 24 h. Statistics: Independent Mann–Whitney (**c**) or dependent Wilcoxon (**d**) test. **n**: cells, **N**: donors.

The cytoplasmic concentration of Ca^2+^ ions is tightly regulated in T cells but can be increased by release from intracellular stores, or by entry through plasma membrane Ca^2+^ ion channels.^36^ A representative live cell-Ca^2+^ recording trace measured from activated CD4^+^ T cells (Fig. 4a) showed that acute GABA (500 nM) application induced a transient calcium signal, which was, in fact, influx of extracellular calcium and not due to release from intracellular stores as no signal could be detected in the absence of extracellular calcium (Fig. 4a). The cells had not been exposed to GABA before. Analysed live cell-Ca^2+^ recording data from four donors is shown in Fig. 4b. What mechanisms the decay of the Ca^2+^ signals is related to, remains to be studied. Transient, acute change in glucose from 5.5 to 16.7 mM did not alter the 500 nM GABA-activated Ca^2+^ response (Supplementary Fig. S4c). Insulin incubation of cells enhanced the physiological, saturating (500 nM) GABA-induced Ca^2+^ signals in 5.5 mM but not in 16.7 mM glucose (Fig. 4c), whereas no differences were observed for baseline Ca^2+^ levels in cells with or without insulin incubation (Supplementary Fig. S4d). The GABA-activated Ca^2+^ signal was blocked by the GABA_A_R antagonist picrotoxin (Fig. 4d), while no effect was observed by the GABA_B_R antagonist, CGP52432 (Supplementary Fig. S4e). This prompted us to examine the dose-response relationship between GABA and the Ca^2+^ signals in cells with or without insulin incubation (Fig. 4e, f, g; Supplementary Fig. S4f and g). Representative time-lapse recording of a CD4^+^ T cell, 16.7 mM glucose, with insulin, demonstrates that raising GABA concentrations induced increasing Ca^2+^ signals (Fig. 4e). Insulin incubation of the cells lowered the EC_50_ values of the GABA-activated Ca^2+^ signals. The apparent GABA affinity of the Ca^2+^ signal was 40 nM and 156 nM GABA in 5.5 mM and in 16.7 mM glucose, and shifted more than 1000-fold to 0.005 nM and 0.14 nM GABA with insulin, respectively (Fig. 4f and g). At high GABA concentrations (≥10 μM), the Ca^2+^ signal decreased which may be related to desensitisation of the GABA_A_R (Supplementary Fig. S4g). The GABA-induced Ca^2+^ response and enhancement by insulin is in accordance with our electrophysiological patch-clamp data (Fig. 3a, b, c). These data demonstrate that physiological GABA (pM—nM) increases Ca^2+^ entry in activated CD4^+^ T cells, and the response is profoundly enhanced by insulin.

### GABA_A_R agonists evoke Ca^2+^ entry in CD4^+^ T cells

**Fig. 5:**
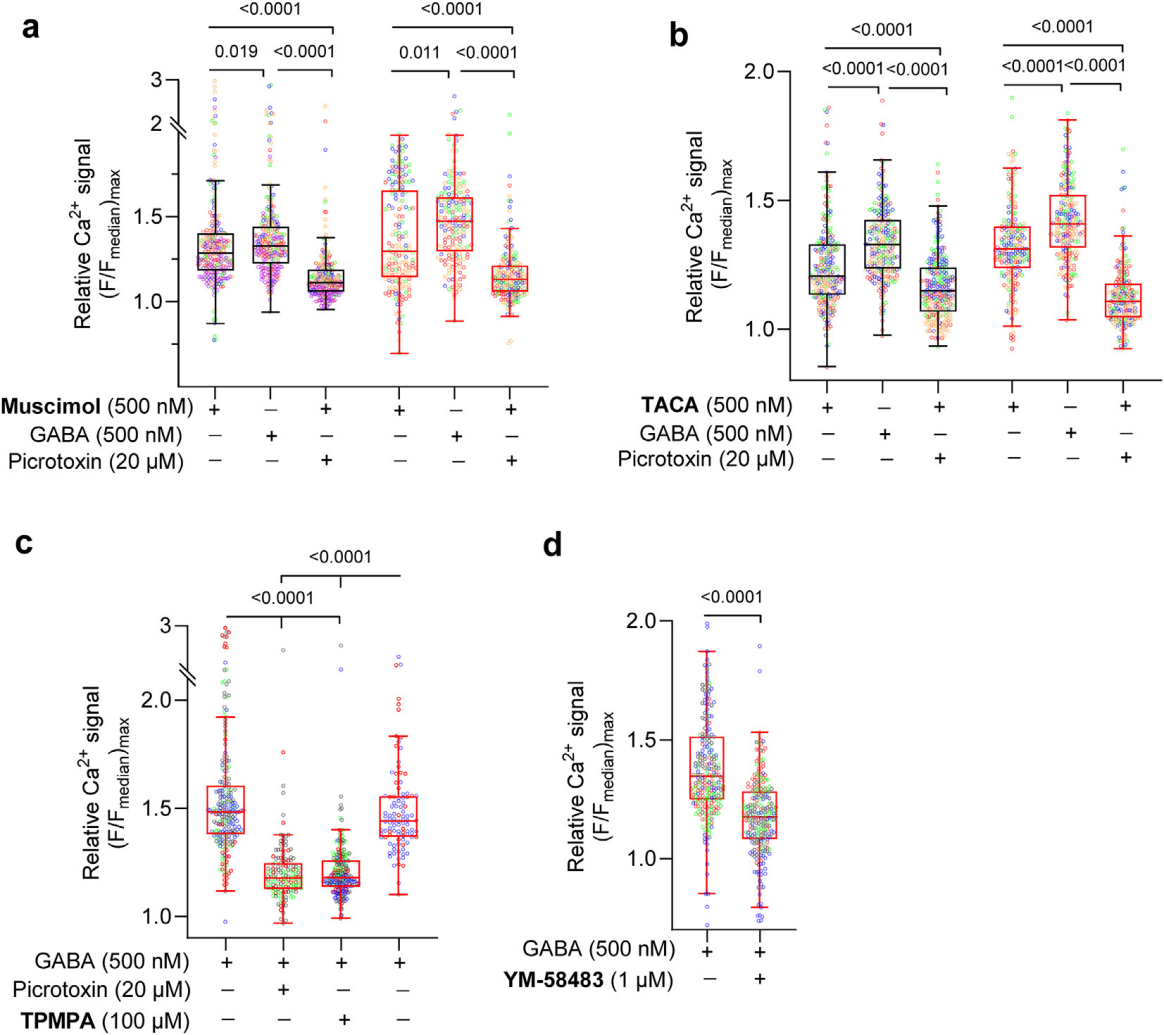
**Pharmacological profile of GABA-activated Ca**^**2+**^**entry in human CD4**^**+**^**T cells**. Effects of GABA_A_R agonists; muscimol (500 nM, **a**) and TACA (500 nM, **b**), GABA_A_R antagonists picrotoxin (PTX, 20 μM, **a, b, c**) and TPMPA (100 μM, **c**), and SOCE antagonist YM58483 (1 μM, **d**) on relative Ca^2+^ signal in activated CD4^+^ T cells in 5.5 mM glucose (n = 191–265, N = 3–5). Individual data point represents relative Ca^2+^ signal recorded from each cell and is color-coded based on donors. Box-whisker plots (black without and red with insulin) display whiskers usingTukey's method. In experiments with insulin, 48 h post-activation insulin was added for 24 h. Statistics: Friedman test followed by Dunn's multiple comparisons test (**a, b, c**) or paired Wilcoxon test (**d**). **n**: cells, **N**: donors.

To further confirm the GABA_A_R connection in the GABA-evoked Ca^2+^ signal, we examined the pharmacological profile of the Ca^2+^ response using GABA_A_R agonists and antagonists. The agonist TACA and the antagonist TPMPA are most selective for ρ-containing GABA_A_R.^37^ In activated CD4^+^ T cells treated with or without insulin, the application of the GABA_A_R agonists muscimol (Fig. 5a), TACA (Fig. 5b) or GABA (Fig. 5a, b, c) increased the relative Ca^2+^ signals, which were then inhibited by the GABA_A_R antagonists picrotoxin (Fig. 5a, b, c) or TPMPA (Fig. 5c). Homomeric glycine-activated receptors can be inhibited by picrotoxin but not native heteromeric receptors.^38^^,^^39^ No effect of picrotoxin was detected on baseline recordings in the electrophysiological or Ca^2+^ imaging experiments, in line with that to date active glycine receptors have not been identified in T cells.^38^ The pharmacological profile of the GABA_A_R is consistent with ρ-containing GABA_A_R being expressed and functional in the activated CD4^+^ T cells. But whether the receptors are homo- or heteromeric ρ2-containing GABA_A_R remains to be determined.

Ca^2+^ release-activated Ca^2+^ (CRAC) channels mediate store-operated calcium entry (SOCE) in T cells that is central to the Ca^2+^ homeostasis and cytoplasmic Ca^2+^ levels.^36^ The GABA-activated Ca^2+^ signal was inhibited by 1 μM YM58483 (Fig. 5d), an antagonist of SOCE, revealing the Ca^2+^ ion channel participating in the cascade. The tight scaling of the Ca^2+^ signal from GABA_A_R activation to Ca^2+^ entry and the sensitisation by insulin identifies a specific, focused mechanism regulating Ca^2+^ signalling in CD4^+^ T cells.

Insulin is known to regulate intracellular signalling and gene expression, whereas less is known about the effects of GABA. However, out of the nine examined genes encoding transcription factors or kinases, only *NFATC2* (nuclear factor of activated T cells 2, NFAT1) was significantly reduced by GABA at 5.6 mM glucose (Supplementary Table S2). At the protein level we observed a decrease with GABA for 10 mM but not for 5.6 mM glucose (Supplementary Fig. S5). Similar results were obtained for NFAT2 but no change was identified for NFAT4 (Supplementary Fig. S5). NFATs proteins are activated by increased Ca^2+^ in the cytoplasm and translocate to the nucleus, where they impact T cell metabolism and regulate the transcription of many proteins including hexokinase and inflammatory molecules.^40^

### GABA regulation of glycolysis is glucose-dependent

**Fig. 6:**
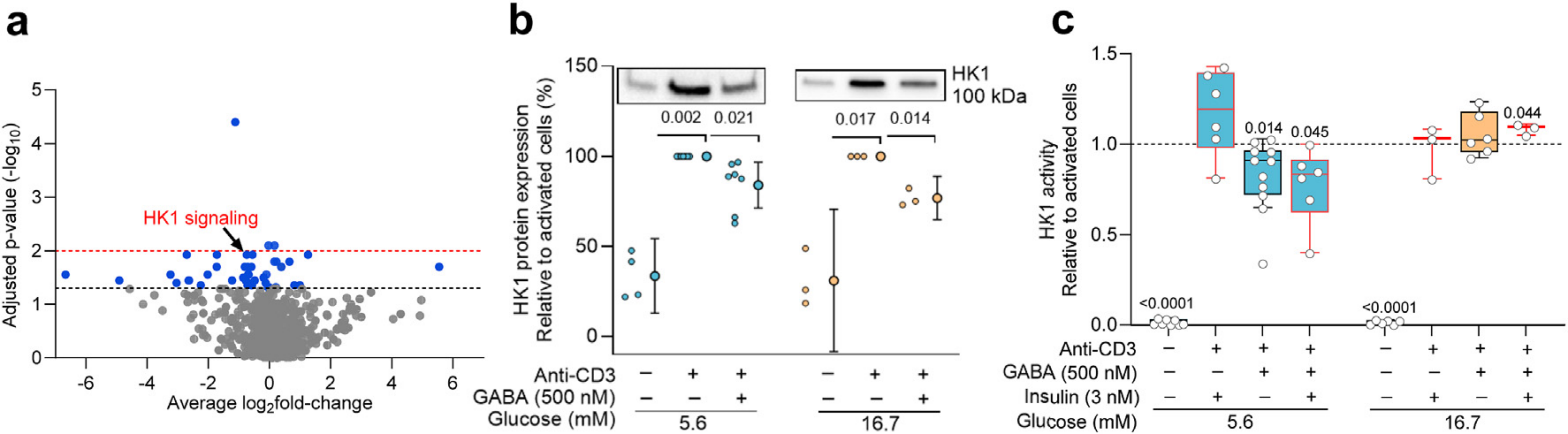
**GABA inhibits hexokinase in activated CD4**^**+**^**T cells**. **a)** The volcano plot summarises pathways that are significantly regulated (blue dots including HK1 signalling) by GABA treatment in cells 72 h post activation (N = 5), 5.6 mM glucose. The x-axis represents the average fold-change of all proteins measured by mass spectrometry within that pathway and every dot is one pathway. The y-axis represents the adjusted P-value (-log10 transformation). The horizontal black and red line represents P = 0.05 and 0.01, respectively. **b)** Immunoblot images and relative expression of HK1 protein (N = 3–7). Target band volumes after total protein normalisation were further normalised to values of activated cells in the absence of GABA at each glucose concentration. Data are presented as mean with 5–95 percentile. **c)** In vitro HK activity analysed in resting and activated cells, 5.6 mM (N = 12) or 16.7 mM (N = 6) glucose. HK activity (μmol NADH/min/ml) was normalised to the activity of activated cells in the absence of drugs. Box-whisker plots (black without and red with insulin) display the whiskers using Tukey's method. In experiments with insulin, 48 h post-activation insulin was added for 24 h. Statistics: one sample t-test when compared to activated cell group, N: donors.

**Fig. 7:**
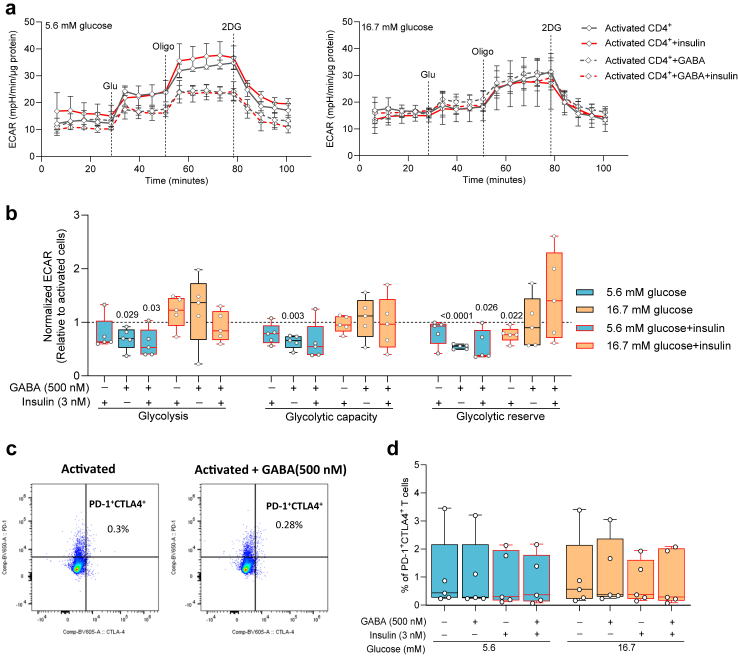
**GABA inhibits glycolysis in activated CD4**^**+**^**T cells**. **a)** Representative ECAR over time in cells 72 h post-activation treated with or without GABA (500 nM) or insulin at 5.6 mM (left panel) or 16.7 mM (right panel). Dashed lines indicate injections into media of glucose (Glu.), oligomycin (Oligo) or 2-deoxyglucose (2-DG). Data represent mean ± SD. **b)** ECAR results (mpH/min/protein) were normalised to controls in absence of drugs for average glycolysis, glycolytic capacity and glycolytic reserve in cells 72 h post-activation treated with or without GABA or insulin, 5.6 mM (N = 5) or 16.7 mM (N = 5) glucose. Data are normalised to values of activated cells in the absence of drugs at each glucose concentration. **c, d)** Representative density plots (**c**) and quantification (**d**) showing the low percentage of PD-1^+^CTLA4^+^ (exhausted) CD4^+^ T cells by flow cytometry. Box-whisker plots (black without and red with insulin) display the whiskers using Tukey's method (**b, d)**. In experiments with insulin, 48-h post-activation insulin was added for 24-h. Statistics: one sample t-test when compared to activated cell group (**b**), Friedman test followed by Dunn's multiple comparisons test (**d**). **N**: donors.

That GABA regulated expression of the signalling pathways and proteins was revealed by mass spectrometry (MS) of activated CD4^+^ T cells cultured in the presence or absence of GABA (Fig. 6a, Supplementary Table S3). Hexokinase 1 (HK1), the first enzyme in the glycolytic pathway, was significantly down-regulated by GABA (Fig. 6a) and in both 5.6 mM and 16.7 mM glucose (Fig. 6b). HK activity was negligible under resting conditions but increased about 60 times in activated CD4^+^ T cells in both 5.6 mM and 16.7 mM glucose. GABA decreased the HK activity but only at 5.6 mM glucose and insulin had no further effect whereas in 16.7 mM glucose, in GABA and insulin, the HK1 activity increased somewhat (Fig. 6c). T cells switch to aerobic glycolysis upon activation as their predominant way to generate adenosine 5′-triphosphate (ATP) and to accumulate biomass,^41^^,^^42^ the so-called Warburg effect. It results in glucose-derived pyruvate being converted to lactate that can be detected by measuring the extracellular acidification rate (ECAR).^26^ Since HK is the gatekeeper for the glycolytic pathway, we examined if glucose, GABA or insulin modulated glycolysis. Representative ECAR traces and average values for activated CD4^+^ T cells at 5.6 mM and 16.7 mM glucose in the presence or absence of GABA and insulin are shown in Fig. 7a and b. GABA but not insulin inhibited glycolysis and glycolytic capacity but only in 5.6 mM glucose. Furthermore, the glycolysis and glycolytic capacity was reduced, in general, about 60% in 16.7 mM as compared to 5.6 mM glucose. No effect of GABA at 16.7 mM glucose might suggest the cells became exhausted or senescent in the high glucose concentration. Flow cytometry analysis of the T cells at 5.6 mM and 16.7 mM glucose revealed very low levels of exhaustion (Fig. 7c and d) and senescence, which were not affected by GABA or insulin treatment (Supplementary Fig. S6). The results are in agreement with a study reporting that hyperglycaemia hampers functionality of human CD8^+^ T cells.^43^ Our results raise the possibility that high intracellular glucose levels negatively influence the GABA inhibition of glycolysis.

## Discussion

**Fig. 8:**
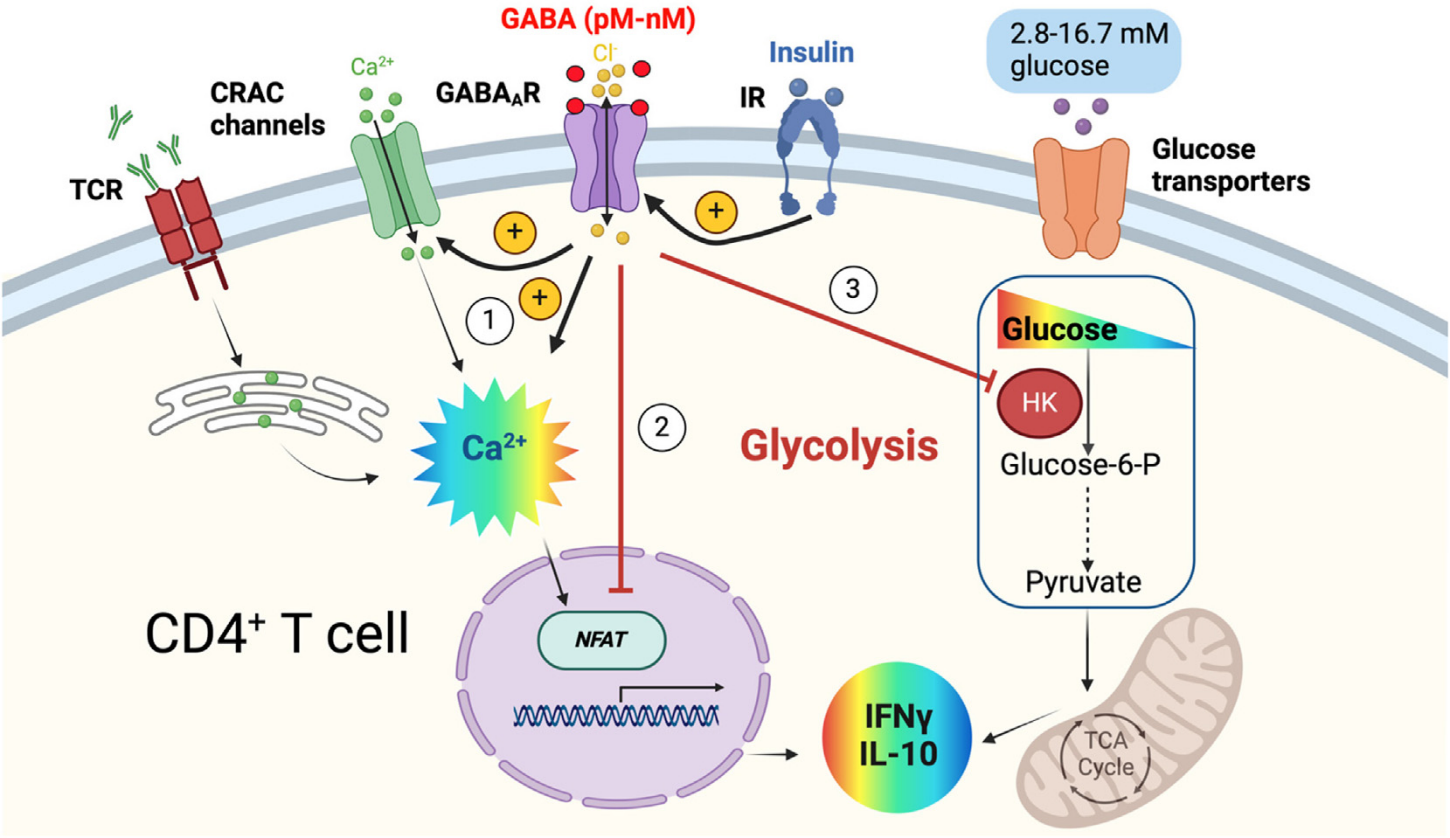
**GABA, glucose and insulin regulate CD4**^**+**^**T cell functions**. In human CD4^+^ T cells, the activation of the T cell receptor (TCR)/CD3 complex by anti-CD3 antibodies binding, initiates store-operated Ca^2+^ entry (SOCE) through Ca^2+^ release-activated Ca^2+^ (CRAC) channels. It regulates intracellular Ca^2+^ signals, drives changes in transcription factors, upregulates glycolysis and increases T cell effector functions. Physiological concentration of GABA (pM—nM) activates GABA_A_R to modulate T cells metabolic activity and levels of released cytokines (e.g., IFNγ and IL-10) by 1) enhancing function of CRAC channels and Ca^2+^ entry; 2) reducing expression of Nuclear Factor of Activated T cell (NFAT); 3) decreasing expression and activity of hexokinase (HK), the 1st rate-limiting enzyme in aerobic glycolysis. In addition, insulin–insulin receptor (IR) signalling reduces glycolysis by enhancing GABA_A_R function. The fluctuation of blood glucose concentration will affect glycolysis and cytokine release from T cells. Created with BioRender.com.

T cells modulate their metabolism to adapt to different environments.^1^^,^^41^^,^^42^ When CD4^+^ T cells are activated, their metabolic activity is altered to meet the demands of cell growth, proliferation and effector functions. Here, we revealed that GABA, glucose and insulin together orchestrate functions of CD4^+^ T cells, to coordinate and to set the activity level of distinct cellular pathways linked to glucose metabolism and that are central for T cells effector functions (Fig. 8).

Proliferating T cells shift their metabolic phenotype to aerobic glycolysis at the expense of mitochondrial oxidative phosphorylation.^41^^,^^44^ Although this switch is inefficient in terms of generating ATP, it confers an advantage for the biosynthetic pathways that branch out from glycolysis.^41^^,^^42^ GABA decreased cellular metabolic activity and the release of proteins in a glucose concentration-dependent manner. GABA_A_Rs are widespread in the brain and are present in many other tissues,^45^ but GABA_A_Rs containing GABRR (ρ subunits) are relatively rare outside the immune system, with the exception of the retina^46^^,^^47^ and so far no pharmacological characterisation of native, human ρ2-containing GABA_A_R has been reported. The functional and pharmacological properties of GABA_A_R receptors are dictated by their subunit composition.^48^ The TACA activation and TPMPA inhibition of the receptors is consistent with ρ2-containing GABA_A_Rs but we do not know if the receptors in T cells are formed as homomeric ρ2 or ρ2 expressed with other GABA_A_R subunits.^47^^,^^49^ The GABA_A_R-ρ2 are abundantly expressed in the CD4^+^ T cells and are upregulated by insulin, suggesting they might be a valuable and rather specific drug target.^47^^,^^50^ Insulin shifted the GABA_A_R signalling by making it supersensitive to GABA, enhancing the GABA-switch, and consequently augmented the Ca^2+^ entry through CRAC channels, an elegant way of modulating intracellular Ca^2+^ levels and intracellular processes affecting T cell functions.

CRAC channel-mediated SOCE regulates several Ca^2+^ signalling downstream pathways, including the expression of glucose transporters and glycolytic enzymes such as HK by activation of NFAT transcriptional factors in T cells.^40^ Our data show that GABA may decrease the expression of NFATs and the expression and enzymatic activity of HK at 5.6 mM glucose, in activated CD4^+^ T cells. Consequently, at the level of HK, there may be a cross-over of GABA signalling and glucose uptake for regulating glycolysis and, thereby, effector functions of T cells. HK is the first enzyme in the glycolytic pathway and by converting glucose to glucose-6-phosphate (G-6-P), traps glucose within the cell. Human HK has normally maximum activity in 5 mM glucose in the presence of natural inhibitors, such as G-6-P,^51^ but HK activity is increased at glucose concentrations >5 mM.^51^ This is in line with our results, where GABA only succeeded decreasing HK enzymatic activity in 5.6 mM glucose. Glucose competes with G-6-P for the binding site on HK,^51^ decreasing the inhibition of HK,^51^ which results in increased substrate availability for biosynthesis as the intracellular glucose concentration increases.^14^ Aerobic glycolysis directly regulates synthesis of IFNγ^52^ by engaging glyceraldehyde-3-phosphate dehydrogenase (GAPDH) and, thereby, relieving its inhibition of IFNγ mRNA translation. In concordance, activated CD4^+^ T cells secreted more IFNγ in high, atypical, glucose concentration. GABA induced a glycolytic shift reducing both glycolysis and the glycolytic capacity values in 5.6 mM glucose, which was absent in 16.7 mM glucose. Discovering that high glucose levels attenuate the immunosuppressive effect of GABA on CD4^+^ T cells, highlights the importance of metabolic factors in the context of GABA immunomodulation. Therefore, a combination treatment with glucose uptake inhibitors and GABA may synergistically dampen T cell functions.

The strength of the study is that it demonstrates how dynamic, environmental factors in the form of physiological molecules can shape the functional response of CD4^+^ T cells. Furthermore, we used human, primary CD4^+^ T cells to study the GABA signalling in immune cells. We and others have previously shown interspecies differences in the pentameric GABA_A_R subunit composition in T cells^49^^,^^53^ and in pharmacology.^47^ For instance, ρ2 and γ2 subunits are only detected in human and mouse T cells, respectively,^49^ and human ρ2-GABA_A_R receptors are much more sensitive to picrotoxin than the rat receptors.^47^ These species differences highlight the importance of using human cells to study functional and pharmacological characteristics of GABA signalling in T cells.

A limitation of our study is the number of samples from patients with T1D that were too few for carrying out detailed analysis of the cells. For the samples obtained from individuals that were healthy, we isolated CD4^+^ T cells from buffy coats prepared by the University hospital blood centre. The individuals that donated blood, self-reported to be healthy and were between 20 and 30 years old. However, other variables, including sex or undetected or unreported inflammation could potentially be confounding factors in this study. It will be critical to characterise further the GABA inhibition of Th1 respective to Th2 cells. So far it appears that GABA inhibits many more pro-inflammatory as compared to anti-inflammatory proteins but the specificities may be related to the microenvironment and other molecules present. It will be important to conduct further studies on the GABA-mediated inhibition of T cell functions in cells from individuals with either type 1 or type 2 diabetes where blood glucose concentrations may be increased above typical, physiological values.

In conclusion, physiological factors such as glucose, GABA, and insulin guide the adaptation of CD4^+^ T cells and modify their functions. The reduction of metabolic activity and the decreased release of inflammatory molecules in response to GABA, revealed a switch that can be turned on or off but a switch that is also modified by insulin and glucose. The study unveils the mechanisms of GABA modulation of CD4^+^ T cells. Understanding the interaction between GABA and metabolic factors may have implications for conditions like diabetes, where immune responses and glucose levels are dysregulated.

## Contributors

BB, ZJ, HH conceived project; HH, ZJ, AKB, SVK, MKM, RSL, BB designed experiments; HH, ZJ, AKB, SVK, OTR, SK, ZG, AIC, FAS, RSL and BB did experiments; ZJ, HH, AKB, SVK, RSL, BB made figures; ZJ, HH, AKB, SVK, OTR, SK, ZG, AIC, FAS, RSL, ETJ, MKM, BB analysed results; ZJ, BB assessed and verified all underlying data; ZJ, HH, AKB, SVK, ETJ, GC, PEA, PB, MKM, BB contributed to project supervision, POC provided samples from patients with T1D, BB wrote manuscript that was edited by ZJ, HH, AKB and then commented on by all other authors. All authors agree with the content of the manuscript. All authors read and approved the final version of the manuscript. BB, ZJ are responsible for decision to submit manuscript.

## Data sharing statement

Proteomics data are available via ProteomeXchange with identifier PXD041654. Other data will be made available on reasonable request by email to the corresponding author.

During the preparation of this work the author(s) used https://chat.lmsys.org/? in order to check correct use of grammar. After using this tool/service, the author(s) reviewed and edited the content as needed and take(s) full responsibility for the content of the publication.

## Declaration of interests

The authors declare no conflict of interest, except that BB has patent applications WO2019/164446A1 and WO2019162403A1.

## Appendix A Supplementary data

Supplementary Figures and Tables

Supplemental Western blots
