# Supplementary material for: GABA-mediated inhibition of human CD4+ T cell functions is enhanced by insulin but impaired by high glucose levels
Source: eBioMedicine. 2024 Jun 28;105:105217. doi: 10.1016/j.ebiom.2024.105217 (PMC11260598; doi:10.1016/j.ebiom.2024.105217)
Supplement: Supplementary Figures and Tables [file mmc1.docx]

**Supplementary Materials for**

**GABA-mediated inhibition of human CD4^+^ T cell functions is enhanced by insulin but impaired by high glucose levels**

Zhe Jin^1^#, Hayma Hammoud^1^#, Amol Keshavasa Bhandage^1^, Sergiy Vasylyovych Korol^1^, Olivia Trujeque-Ramos^1^, Stasini Koreli^1^, Zhitao Gong^1^, Azasul Islam Chowdhury^1^, Friederike Andrea Sandbaumhüter^2^, Erik Tomas Jansson^2^, Robin Sean Lindsay^1^, Gustaf Christoffersson^1^, Per Erik Andrén^3^, Per-Ola Carlsson^1^, Peter Bergsten^1^, Masood Kamali-Moghaddam^3^, Bryndis Birnir^1^*

^1^ Department of Medical Cell Biology, Uppsala University, Uppsala, Sweden

^2^ Department of Pharmaceutical Biosciences, Uppsala University, Uppsala, Sweden.

^3^ Department of Pharmaceutical Biosciences, Spatial Mass Spectrometry, Science for Life Laboratory, Uppsala University, Uppsala, Sweden.

^4^ Department of Immunology, Genetics and Pathology, Science for Life Laboratory, Uppsala University, Uppsala, Sweden

**# Contributed equally**

*** Correspondence**

Prof Bryndis Birnir

Department of Medical Cell Biology

Uppsala University

Uppsala, 75124, Sweden

Email: [bryndis.birnir@mcb.uu.se](mailto:bryndis.birnir@mcb.uu.se)

Tel: +46765620243

**Caption for supplementary material**

**Supplementary Figure 1. Schematic diagram of the experimental design.**

**Supplementary Figure 2.** (related to Fig. 1)
**Supplementary Figure 3.** (related to Fig. 2)
**Supplementary Figure 4.** (related to Fig. 3 and 4)
**Supplementary Figure 5.** (related to Fig. 7)
**Supplementary Figure 6.** (related to Results)
**Supplementary Table 1.** Donors‘ demographic information
**Supplementary Table 2.** Relative gene expression of key transcription factors and kinases

**Supplementary Table 3.** Reactome analysis of MS data and related to Fig. 6

**Supplementary Table 4.** List of primers for qPCR analysis

**Supplementary Fig. 1: Schematic diagram of the experimental design.**

**
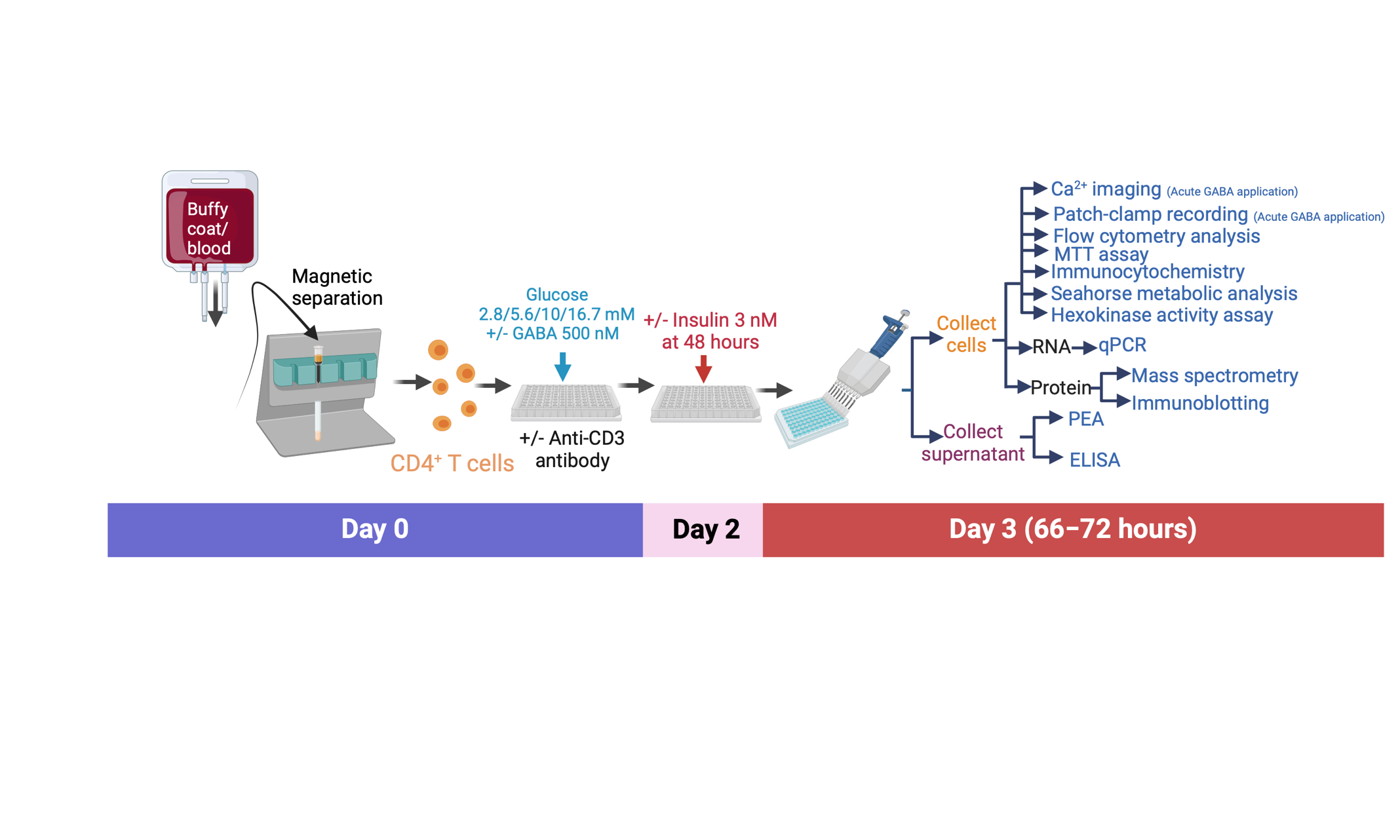
**

**Supplementary Fig. 2: The effect of GABA and glucose on cellular metabolic activity and release of IFNγ and IL-10 from CD4^+^ T cells.**

**a.** Tukey pair-wise comparison of the cellular metabolic activity of CD4^+^ T cells 72 h after activation as measured by MTT assay, in the absence and presence of GABA at different glucose concentrations (N= 32 for 2.8 and 16.7 mM glucose, N= 51 for 5.6 and 16.7 mM glucose). Differences in respected group comparisons are presented as mean with 95% confidence interval. Data were normalized to values of activated cells in the absence of GABA at 5.6 mM glucose concentration. **b**. Distribution (histogram and Gaussian fit) of normalized cellular metabolic activity of CD4^+^ T cells at low normal (2.8 and 5.6 mM) and high (10 and 16.7 mM) glucose concentrations. Solid lines: Gaussian fits, vertical broken line: 10% inhibition by GABA (500 nM). X-axis: normalized cellular metabolic index, Y-axis: relative frequency represented as fractions. **c, d**. Violin plots show metabolic activity of activated CD4^+^ T cells from donors where >10% (**c**) or <10% (**d**) GABA inhibition was observed at 5.6 mM glucose; 2.8 (N=28;4), 5.6 (N=37;14), 10 (N=27;5) and 16.7 (N=36;15), respectively. Horizontal black and white lines indicate the median and quartiles, respectively. **e, f**. IFNγ (**e**) and IL-10 (**f**) released from CD4^+^ T cells 72 h after activation in presence of different glucose concentrations.

Data were normalized to values of activated cells in the absence of drugs at each glucose concentration (**c**, **d**, **e, f**). Box and whisker (**e**, **f**): box as 25-75 percentiles, whiskers determined with Tukey’s method, black lines in the boxes as median. Statistics: Repeated measures two-way ANOVA (mixed model) followed by Tukey for multiple comparisons (**a**), mixed-effects analysis followed by Tukey test for normalized values among different glucose concentrations (**c, d, e, f**). N: donors.


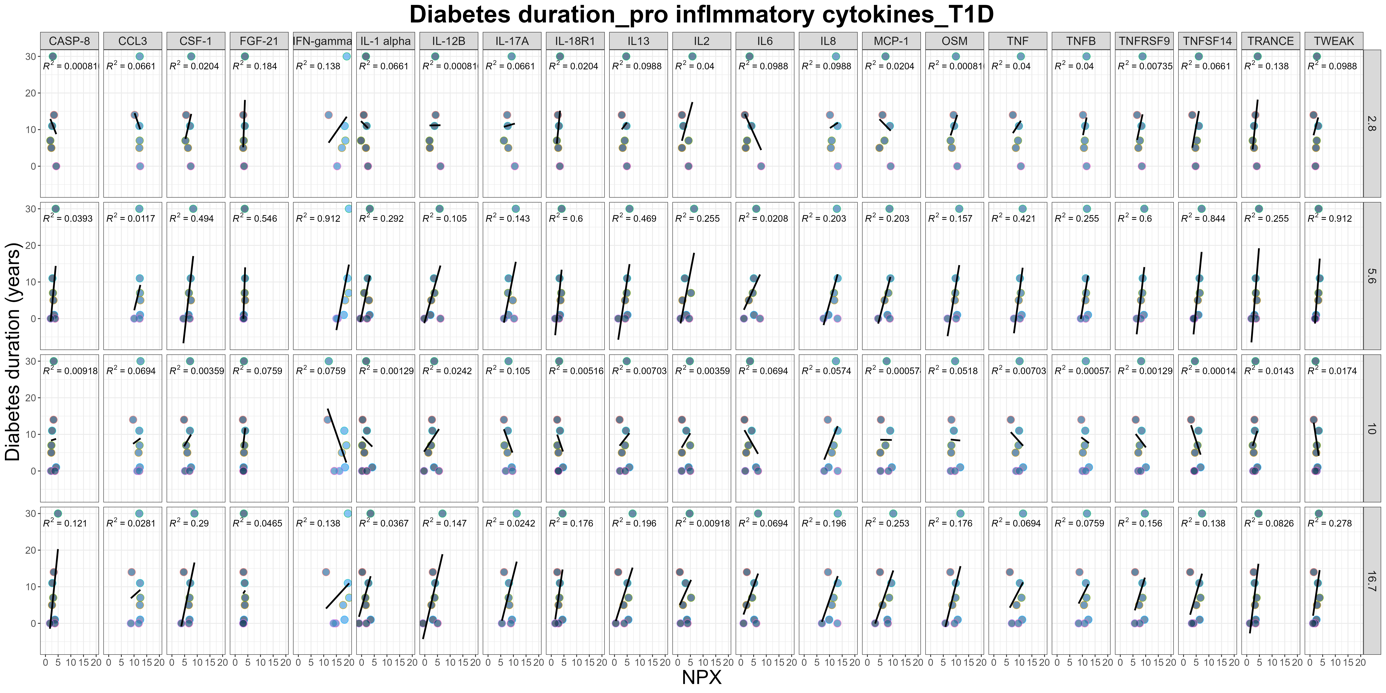


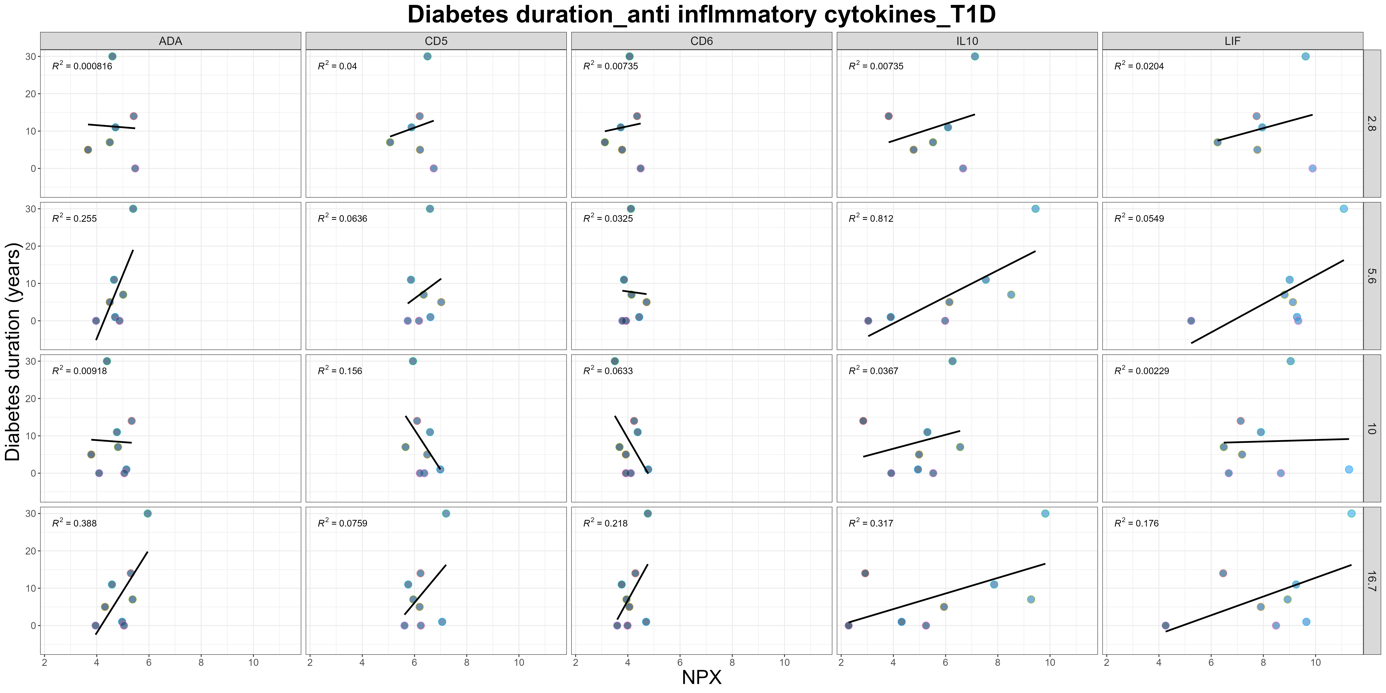


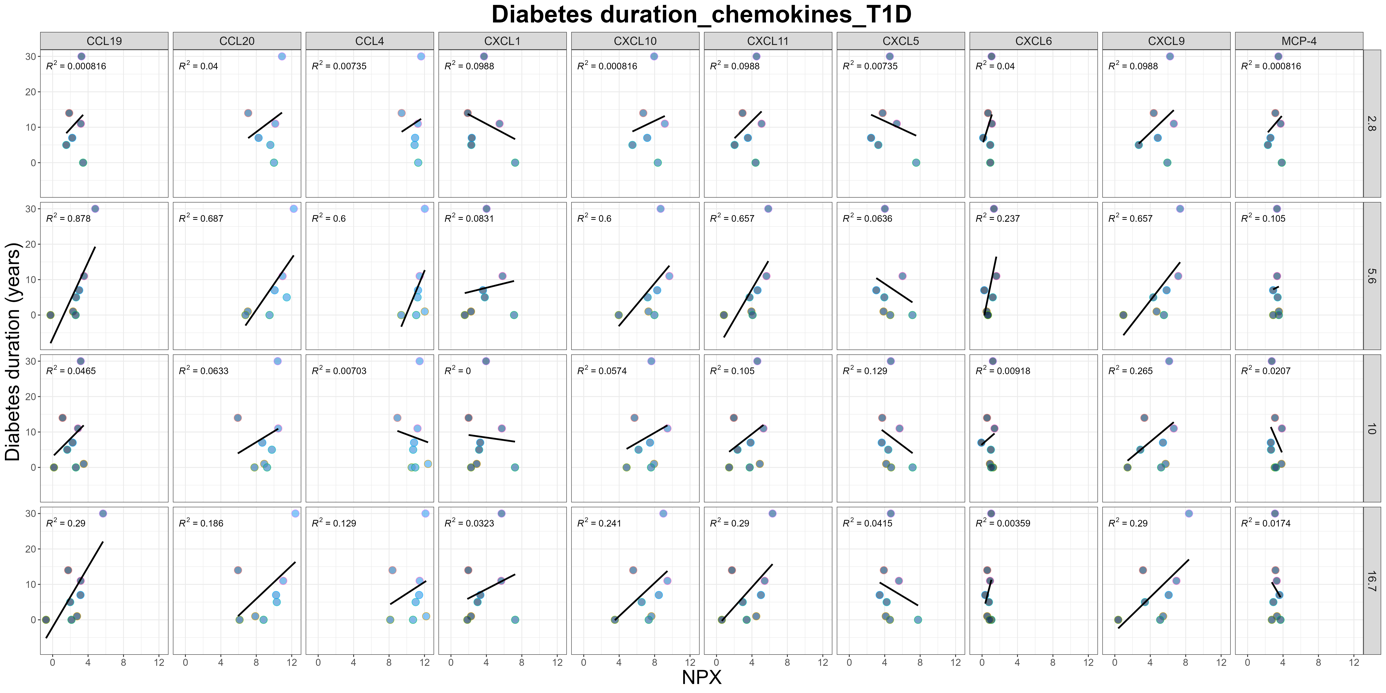


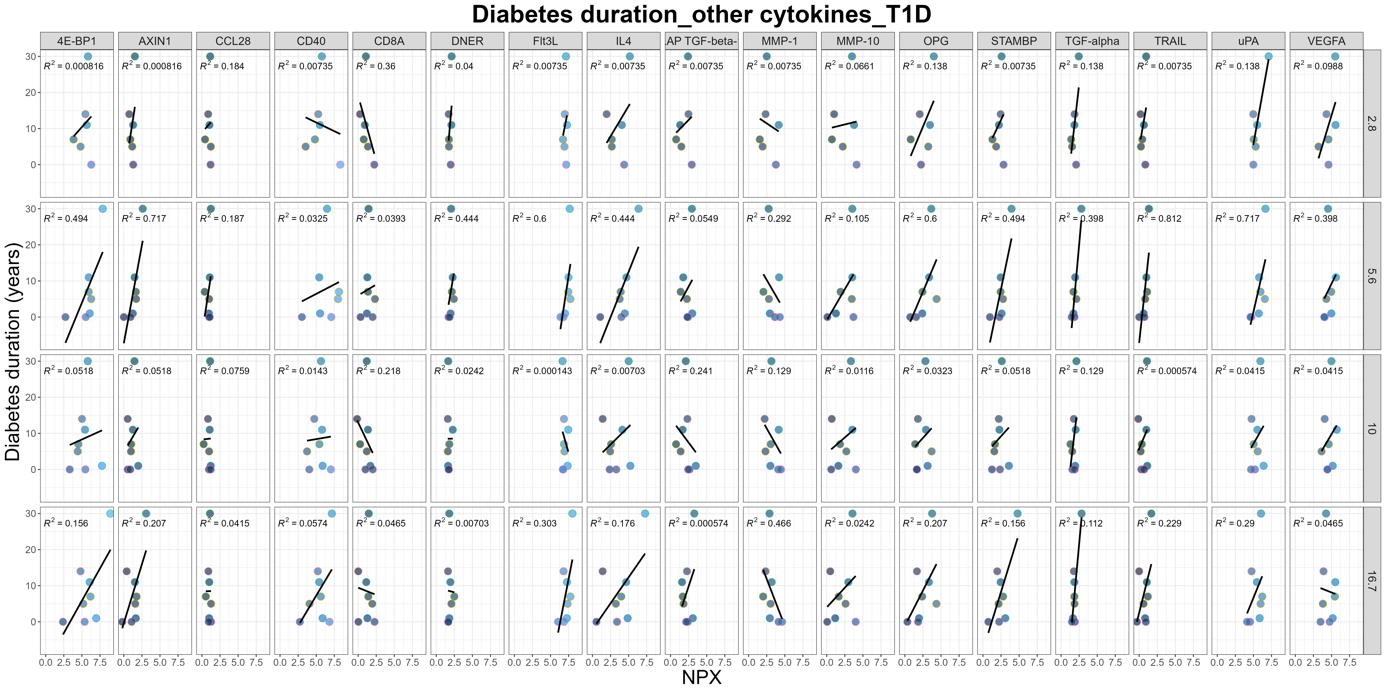


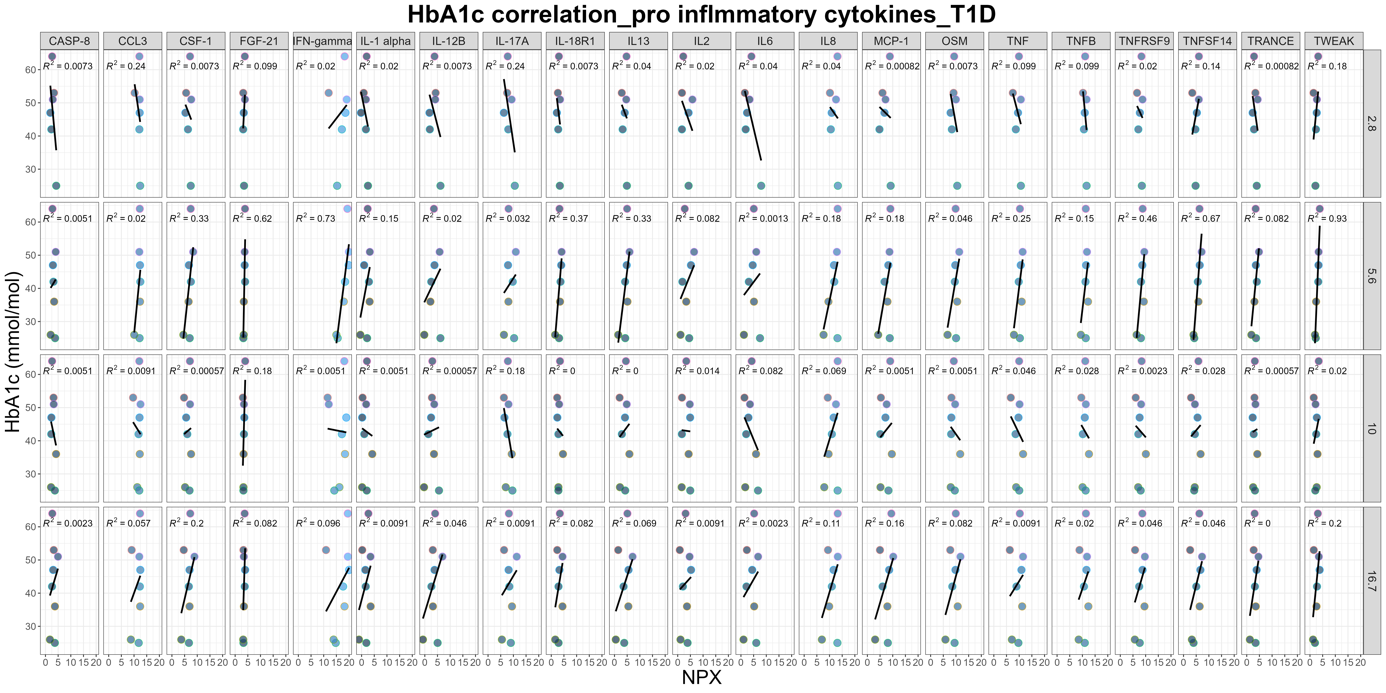


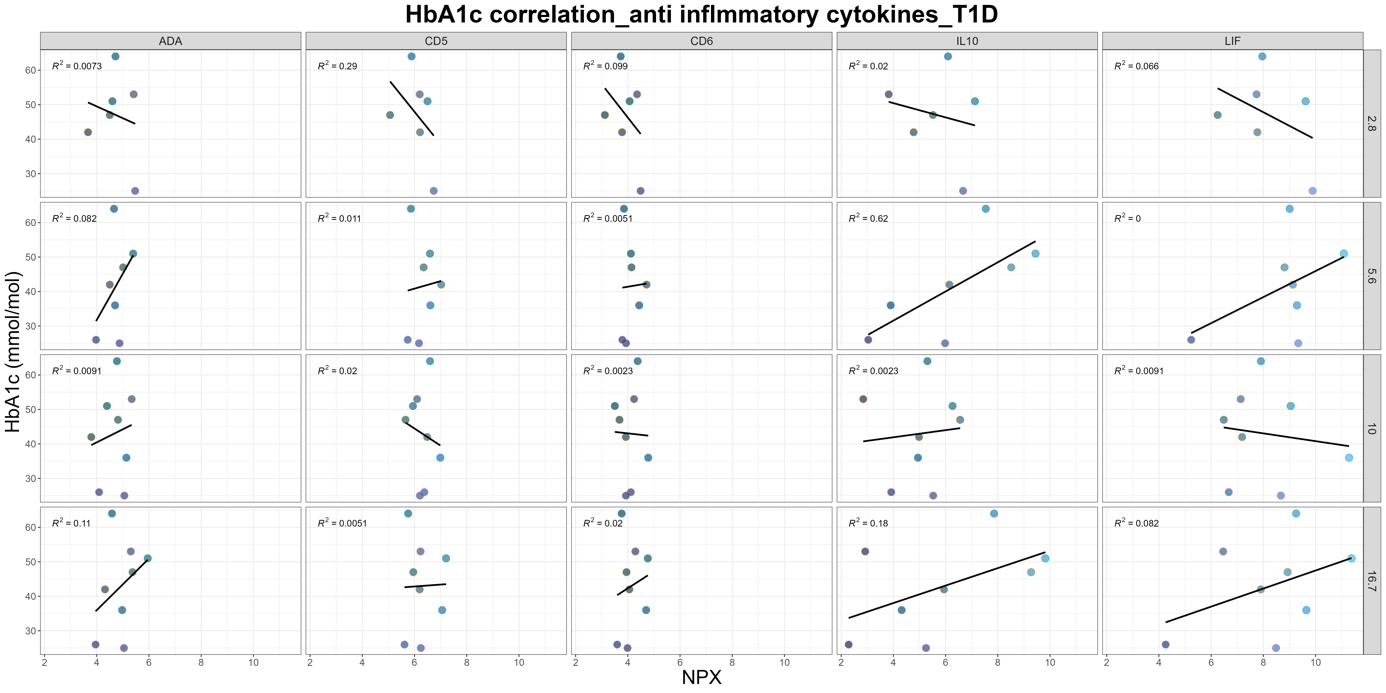


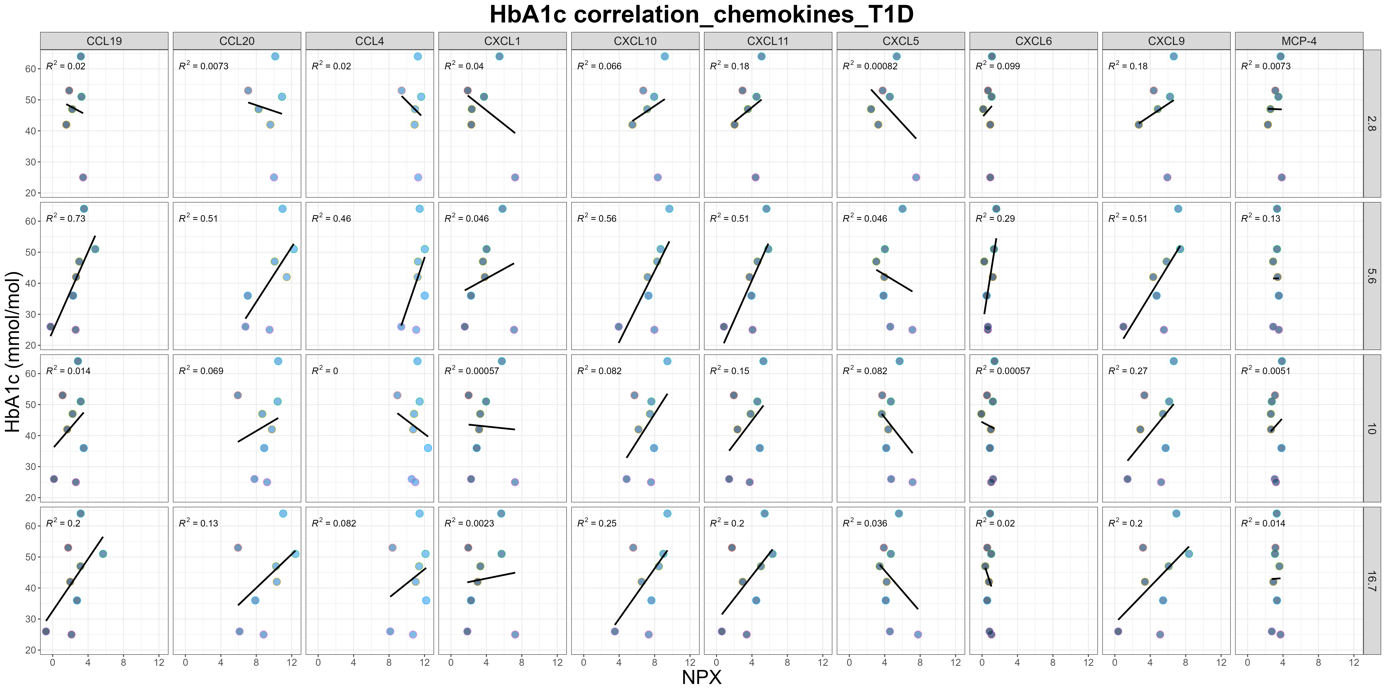


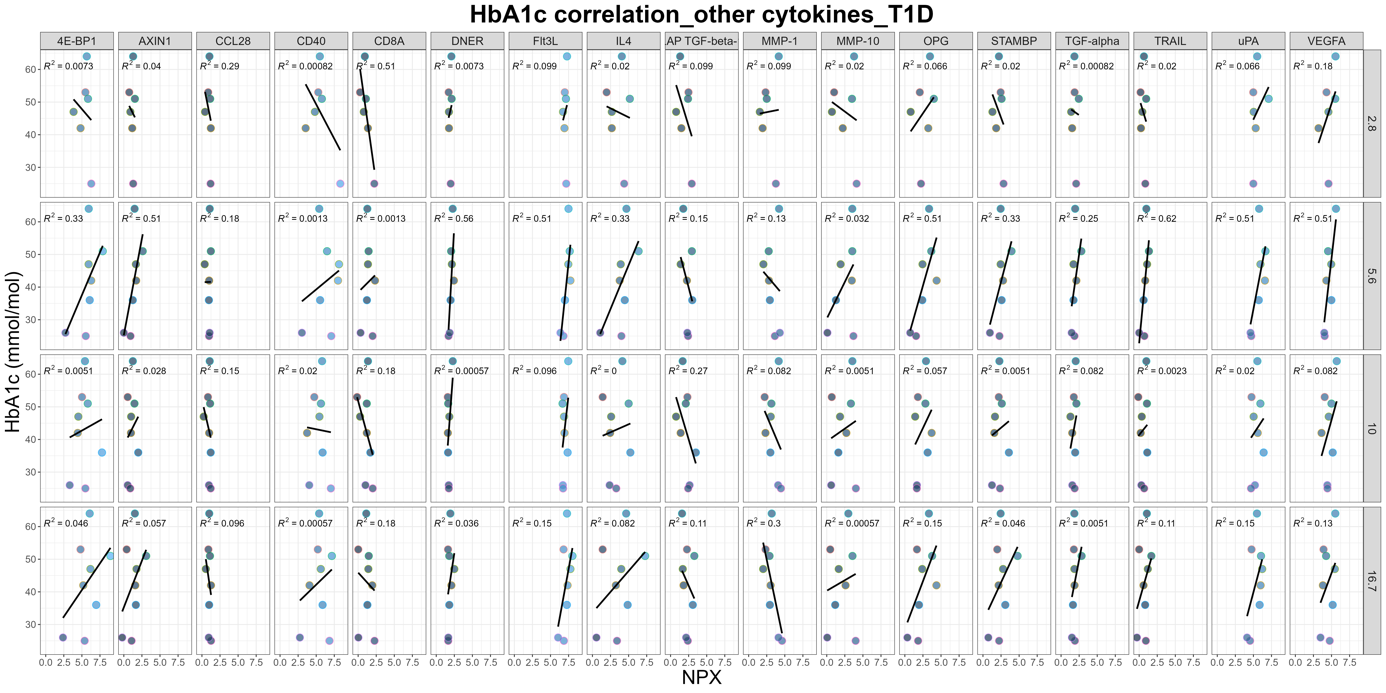


**Supplementary Fig. 3: Correlation between levels of inflammatory-related proteins in cell culture supernatant and diabetes duration or HbA_1c_ of T1D patients.**

The correlation between normalized protein expression (NPX) values from the PEA assay for pro- or anti-inflammatory cytokines, chemokines and other cytokines and diabetes duration (in years) or HbA_1c_ (mmol/mol) of T1D patients (N=8) was assessed by non-parametric Spearman rank correlation.

**Supplementary Fig. 4: GABA-activated current and Ca^2+^ signaling in CD4^+^ T cells.**

**a**. A representative current-voltage relationship recorded in an activated CD4^+^ T cell in response to 500 nM GABA application during perforated patch-clamp, 5.6 mM glucose. **b.** The time course of the GABA_A_R-mediated current under continuous application of 500 nM GABA. The current values are recorded every 25 s at +30 mV. Each circle represents a mean value from n = 4 recordings from three donors, the bars represent SD. **c.** GABA (500 nM)-activated relative Ca^2+^ signals (left panel) and mean intensity (right panel) recorded from activated CD4^+^ T cells (n=128 cells/ N=3 donors) in sequentially applied 5.5 mM and 16.7 mM glucose-containing media. Cells were activated in 5.5 mM glucose-containing media for 72 h. **d.** The ratio of Ca^2+^ signals in the absence and presence of calcium-ionophore calcimycin (1 μM) in CD4^+^ T cells (n=85 cells/ N=4 donors) 72 h after activation with or without insulin treatment. **e.** GABA-activated relative Ca^2+^ signal recorded in CD4^+^ T cells (n=131 cells/ N=3 donors) in the absence or presence of GABA_B_ receptor antagonist CGP 52432 (1 µM) at 5.5 mM glucose. **f.** Relative Ca^2+^ signals induced by different concentrations of GABA in activated CD4^+^ T cells treated without or with insulin in 5.5 mM or 16.7 mM glucose. **g.** Relative Ca^2+^ signals induced by high GABA concentrations (0.5, 10, 1000 μM), in activated CD4^+^ T cells treated without or with insulin in 5.5 mM or 16.7 mM glucose. Box and whisker (**c**, **d, e**, **f**): box as 25-75 percentiles, whiskers determined with Tukey’s method, black lines in the boxes as median Statistics: Mann-Whitney test (**c, d, e, f**) and two-tailed independent t-test (**g**).

**Supplementary Fig. 5: Flow cytometry analysis of CD4^+^ T cells.**

**a.** Flow Cytometry gating strategy. Example flow plots are from activated T cells in the presence of 5.6 mM glucose from one donor. Following linear unmixing samples were gated to identify CD4^+^ T cells by: singlet, lymphocyte, live, CD3^+^, CD8^-^ CD4^+^. Following CD4^+^ T cell gating samples were gated and quantified for: **b.** CD25^+^FoxP3^-^ (early activated T cells) and CD25^+^FoxP3^+^ (regulatory T cells). **c.** PD-1^+^ CTLA-4^-^ (early activated T cells) and PD-1^+^ CTLA-4^+^ (Exhausted T cells, see Fig. 7c, d). **d.** HLA-DR^+^ (late activated T cells). **e.** Ki67 isotype control and Ki67^+^ (proliferating T cells). **f.** CD57^+^ (Senescent T cells). N=4-5. Activated CD4^+^ T cells were treated with GABA (500 nM) in the absence or presence of glucose (5.6 or 16.7 mM). In experiments with insulin, 48 h post-activation insulin was added for 24 h. Box-whisker plots (black without and red with insulin) display the whiskers using Tukey’s method (**b-f**). Statistics: Friedman test followed by Dunn’s multiple comparisons test (**b-f**).

**Supplementary Fig. 6: NFAT protein expression in human CD4^+^ T cells.**

Representative immunoblot images and relative expression of NFAT1 **(a)**, NFAT2 **(b)** and NFAT4 **(c)**. Target band volumes after total protein normalization were further normalized to values of activated cells in the absence of GABA at each glucose concentration. Data are presented as mean with 5-95 percentile.

**Supplementary Table 1. Donors’ demographic information**

| **Parameter** | **Normal donors** | **Type-1 diabetic donors** |
| --- | --- | --- |
| Age (years) | 27 (24-29) | 27.5 (22-32) |
| Sex |  |  |
| Male | 91/165 (55.2%) | 5/8 (62.5%) |
| Female | 74/165 (44.8%) | 3/8 (37.5%) |
| Age at onset of T1D (years) |  | 16.5 (11-28) |
| Disease duration (years) |  | 6.5 (2-14) |
| GAD positive |  | 5/8 (62.5%) |
| HbA_1c_ (mmol/mol) |  | 52 (44-61) |
| **Descriptive variables are presented as median (IQR), and categorical variables are presented as frequency (percentage).** | | |

**Supplementary Table 2. Relative gene quantification (normalized to activated cells) by qPCR (N=7-11 donors).**

| **Gene name** | **5.6 mM glucose** | | | | | **16.7 mM glucose** | | | | |
| --- | --- | --- | --- | --- | --- | --- | --- | --- | --- | --- |
|  | **Resting** | **Activated** | **Activated+Insulin** | **GABA** | **GABA+Insulin** | **Resting** | **Activated** | **Activated+Insulin** | **GABA** | **GABA+Insulin** |
| *NFATC1* | 0.088 | 1^***^ | 1.129^***^ | 1.086^**^ | 1.140^***^ | 1.902 | 1^**^ | 1.017 | 1.072^*^ | 0.923^**^ |
| *NFATC2* | 0.996 | 1 | 0.980 | 0.884^##^ | 0.942 | 1.333 | 1 | 0.961^&^ | 1.121 | 0.876^*, &^ |
| *NFATC3* | 0.512 | 1^**^ | 0.929^**^ | 0.836^*^ | 0.773^**^ | 0.484 | 1^*^ | 0.895^*, &^ | 1.057^**^ | 0.922^*^ |
| *JAK1* | 1.808 | 1^***^ | 1.156 | 1.096^***^ | 1.144^***, #^ | 2.123 | 1 | 1.049 | 1.329 | 1.027 |
| *JAK2* | 0.405 | 1^***^ | 1.060^***^ | 1.085^***^ | 1.133^***^ | 0.291 | 1 | 0.802 | 1.041 | 0.932 |
| *TYK2* | 1.639^#^ | 1 | 1.278 | 1.016 | 0.997^*^ | 1.254 | 1 | 0.716 | 0.826 | 0.596 |
| *HIF1A* | 0.310 | 1^***^ | 1.005^***^ | 0.961^***^ | 0.960^***^ | 0,287 | 1^***^ | 0.919^***^ | 0.968^***^ | 0.794^***^ |
| *STAT1* | 0.088 | 1^***^ | 1.129^***, #^ | 1.086^***^ | 1.140^***^ | 0.067 | 1^***^ | 0.939^***^ | 0.925^***^ | 0.981^***^ |
| *MYC* | 0.626 | 1^**^ | 0.925^**^ | 0.918^*^ | 0.946^**^ | 0.368 | 1^**^ | 0.662^#^ | 0.805^*^ | 0.787^*^ |

*p < 0.05, **p < 0.01, ***p < 0.001, as compared to resting cells; #p < 0.05, ##p < 0.01, as compared to activated cells: &p < 0.05, as compared to GABA-treated cells. The statistical analysis was performed on ∆Cq values with repeated measures one-way ANOVA with Fisher's LSD test for multiple comparison.

**Supplementary Table 3. Reactome analysis of MS data (N=5 at 5.6 mM glucose).**

**Top up-regulated proteins**

| **Identifier** | **logFC** | **AveExpr** | **P.Value** |
| --- | --- | --- | --- |
| K2C79_HUMAN | 0.67 | 13.29 | 0.043 |
| CHM4A_HUMAN | 0.56 | 13.71 | 0.054 |
| HLAH_HUMAN | 1.03 | 14.81 | 0.071 |
| RAB2A_HUMAN | 1.37 | 14.18 | 0.071 |
| SC11A_HUMAN | 5.7 | 10.59 | 0.087 |
| K22O_HUMAN | 0.49 | 12.25 | 0.092 |
| DYL1_HUMAN | 5.55 | 10.78 | 0.10 |
| PLXA4_HUMAN | 0.69 | 13.71 | 0.12 |
| RL36_HUMAN | 6.02 | 12.32 | 0.13 |
| IF4G2_HUMAN | 0.83 | 13.48 | 0.13 |

**Top down-regulated proteins**

| **Identifier** | **logFC** | **AveExpr** | **P.Value** |
| --- | --- | --- | --- |
| TBB1_HUMAN | -7.56 | 8.58 | 0.022 |
| STA5B_HUMAN | -7.61 | 6.04 | 0.037 |
| EXOS3_HUMAN | -6.60 | 5.67 | 0.058 |
| BGAL_HUMAN | -6.67 | 5.78 | 0.061 |
| AL1B1_HUMAN | -6.68 | 5.87 | 0.066 |
| HXK1_HUMAN | -0.74 | 14.37 | 0.068 |
| HLAG_HUMAN | -6.35 | 5.64 | 0.071 |
| COPG1_HUMAN | -5.16 | 10.05 | 0.10 |
| BCL2_HUMAN | -4.91 | 9.87 | 0.12 |
| PIP_HUMAN | -4.68 | 9.68 | 0.13 |

**Supplementary Table 4: Primers for qPCR.**

| **Gene name** | **Primer sequence** |
| --- | --- |
| *STAT1* | Forward 5΄- ATGGCAGTCTGGCGGCTGAATT -3΄  Reverse 5΄- CCAAACCAGGCTGGCACAATTG – 3΄ |
| *MYC* | Forward 5΄- CCTGGTGCTCCATGAGGAGAC - 3΄  Reverse 5΄- CAGACTCTGACCTTTTGCCAGG - 3΄ |
| *JAK1* | Forward 5΄- GAGACAGGTCTCCCACAAACAC - 3΄  Reverse 5΄- GTGGTAAGGACATCGCTTTTCCG -3΄ |
| *JAK2* | Forward 5΄- CCAGATGGAAACTGTTCGCTCAG -3΄  Reverse 5΄- GAGGTTGGTACATCAGAAACACC – 3΄ |
| *TYK2* | Forward 5΄- GGTTGACCAGAAGGAGATCACC – 3΄  Reverse 5΄- TCCTCGTCATCCATCTTGCCCT – 3΄ |
| *HIF1A* | Forward 5΄- AAAATCTCATCCAAGAAGCC - 3΄  Reverse 5΄- AATGTTCCAATTCCTACTGC - 3΄ |
| *NFATC1* | Forward 5΄- CATTTCGGAATCAGAGGATAAC - 3΄  Reverse 5΄- TTATAATTGGAACGTTGGCG - 3΄ |
| *NFATC2* | Forward 5΄- CCAACATGCTTTTTGTTGAG - 3’  Reverse 5΄- TTCGTTTTCTCTTCCCATTG - 3’ |
| *NFATC3* | Forward 5΄- AGAATCTAGAGGTGAACGAG - 3’  Reverse 5΄- CCATCAGATCTTCCTAAATCC - 3’ |
| *IPO8* | Forward 5΄- GCAAAGGAAGGGGAATTGAT - 3’  Reverse 5΄- CGAAGCTCACTAGTTTTGACCC - 3’ |
