# Supplementary material for: GABA-mediated inhibition of human CD4+ T cell functions is enhanced by insulin but impaired by high glucose levels
Source: eBioMedicine. 2024 Jun 28;105:105217. doi: 10.1016/j.ebiom.2024.105217 (PMC11260598; doi:10.1016/j.ebiom.2024.105217)
Supplement: Supplemental Western blots [file mmc2.pptx]

## Slide 1
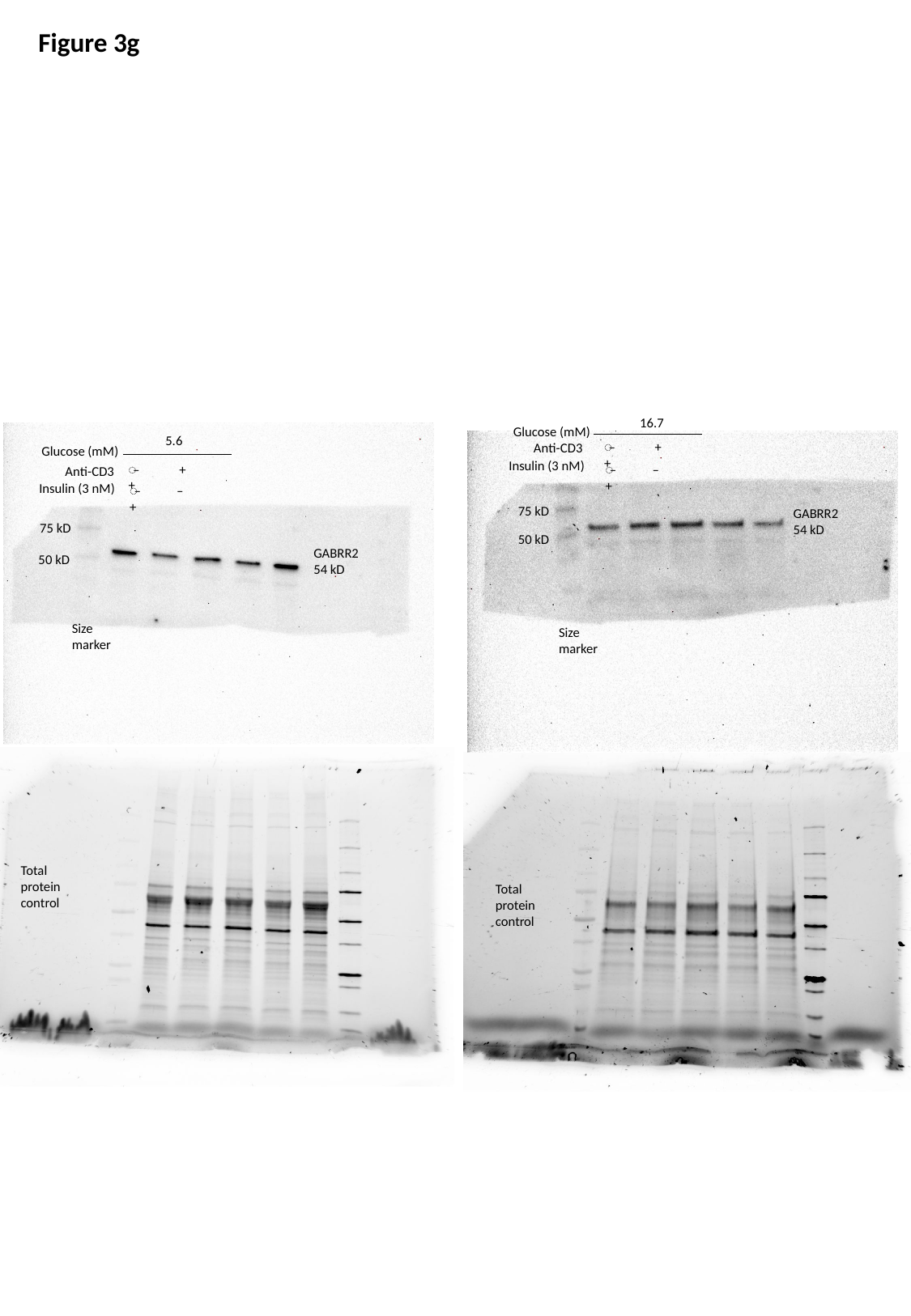

Figure 3g
16.7
Glucose (mM)
5.6
̶ + +
Anti-CD3
Glucose (mM)
Insulin (3 nM)
̶ + +
̶ ̶ +
Anti-CD3
Insulin (3 nM)
̶ ̶ +
75 kD
75 kD
GABRR2
54 kD
50 kD
50 kD
GABRR2
54 kD
Size marker
Size marker
Total protein control
Total protein control

## Slide 2
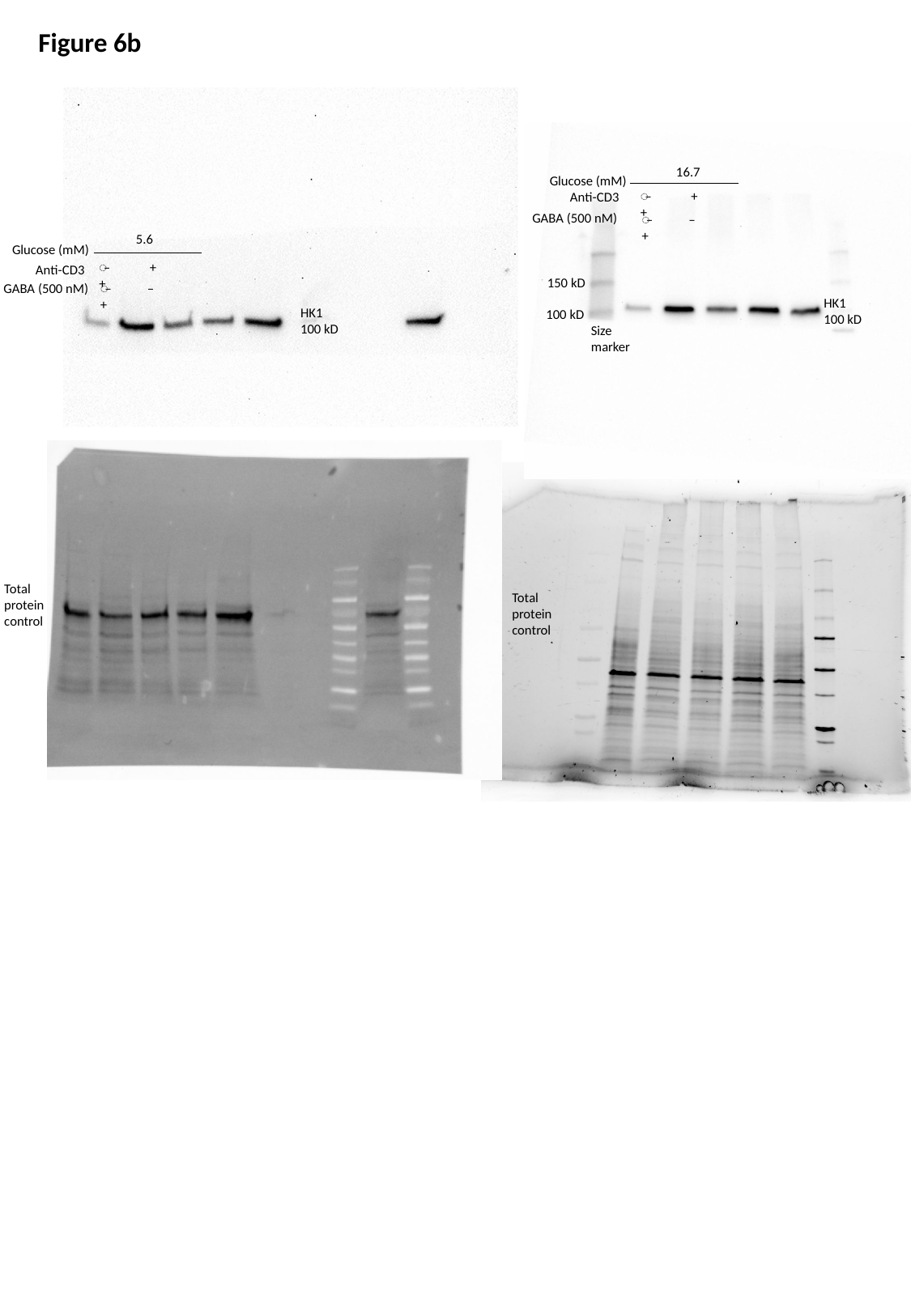

Figure 6b
16.7
Glucose (mM)
̶ + +
Anti-CD3
GABA (500 nM)
̶ ̶ +
5.6
Glucose (mM)
150 kD
̶ + +
Anti-CD3
̶ ̶ +
GABA (500 nM)
100 kD
HK1
100 kD
HK1
100 kD
Size marker
Total protein control
Total protein control

## Slide 3
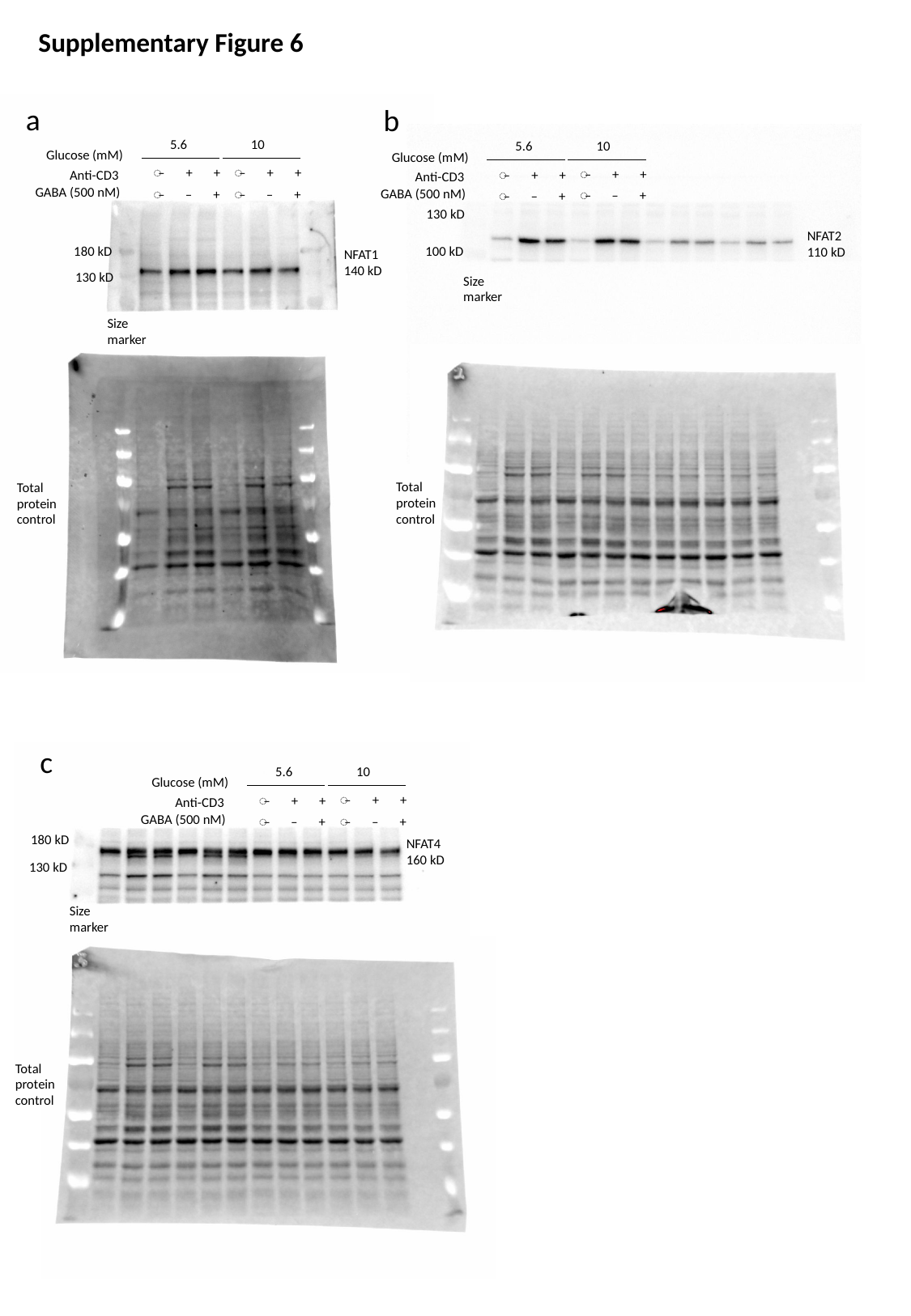

Supplementary Figure 6
a
b
10
5.6
10
5.6
Glucose (mM)
Glucose (mM)
̶ + +
̶ + +
̶ + +
̶ + +
Anti-CD3
Anti-CD3
GABA (500 nM)
GABA (500 nM)
̶ ̶ +
̶ ̶ +
̶ ̶ +
̶ ̶ +
130 kD
180 kD
100 kD
NFAT2
110 kD
NFAT1
140 kD
130 kD
Size marker
Size marker
Total protein control
Total protein control
c
10
5.6
Glucose (mM)
̶ + +
̶ + +
Anti-CD3
GABA (500 nM)
̶ ̶ +
̶ ̶ +
180 kD
NFAT4
160 kD
130 kD
Size marker
Total protein control
